# Seeding activity of human superoxide dismutase 1 aggregates in familial and sporadic amyotrophic lateral sclerosis postmortem neural tissues by real-time quaking-induced conversion

**DOI:** 10.1007/s00401-024-02752-8

**Published:** 2024-06-17

**Authors:** Justin K. Mielke, Mikael Klingeborn, Eric P. Schultz, Erin L. Markham, Emily D. Reese, Parvez Alam, Ian R. Mackenzie, Cindy V. Ly, Byron Caughey, Neil R. Cashman, Moses J. Leavens

**Affiliations:** 1 Department of Biomedical Sciences, McLaughlin Research Institute, 1520 23rd St. South, Great Falls, MT 59405 USA; 2 Center for Biomolecular Structure and Dynamics, University of Montana, 32 Campus Drive ISB #106, Missoula, MT USA; 3 Laboratory of Neurological Infections and Immunity, Rocky Mountain Laboratories, National Institute of Allergy and Infectious Diseases, National Institutes of Health, 903 S. 4th St., Hamilton, MT 59840 USA; 4 Departments of Pathology and Medicine, University of British Columbia, 2211 Wesbrook Mall, Vancouver, BC V6T 2B5 Canada; 5 Department of Neurology, Washington University, 660 S. Euclid Ave, Saint Louis, MO 63110 USA

**Keywords:** Superoxide dismutase 1, Amyotrophic lateral sclerosis, Protein aggregation, Sporadic ALS, Familial ALS, Real-time quaking-induced conversion

## Abstract

Amyotrophic lateral sclerosis (ALS) is a rapidly progressive neurodegenerative disease with average lifespan of 2–5 years after diagnosis. The identification of novel prognostic and pharmacodynamic biomarkers are needed to facilitate therapeutic development. Metalloprotein human superoxide dismutase 1 (SOD1) is known to accumulate and form aggregates in patient neural tissue with familial ALS linked to mutations in their *SOD1* gene. Aggregates of SOD1 have also been detected in other forms of ALS, including the sporadic form and the most common familial form linked to abnormal hexanucleotide repeat expansions in the Chromosome 9 open reading frame 72 (*C9ORF72*) gene. Here, we report the development of a real-time quaking-induced conversion (RT-QuIC) seed amplification assay using a recombinant human SOD1 substrate to measure SOD1 seeding activity in postmortem spinal cord and motor cortex tissue from persons with different ALS etiologies. Our SOD1 RT-QuIC assay detected SOD1 seeds in motor cortex and spinal cord dilutions down to 10^–5^. Importantly, we detected SOD1 seeding activity in specimens from both sporadic and familial ALS cases, with the latter having mutations in either their *SOD1* or *C9ORF72* genes. Analyses of RT-QuIC parameters indicated similar lag phases in spinal cords of sporadic and familial ALS patients, but higher ThT fluorescence maxima by *SOD1* familial ALS specimens and sporadic ALS thoracic cord specimens. For a subset of sporadic ALS patients, motor cortex and spinal cords were examined, with seeding activity in both anatomical regions. Our results suggest SOD1 seeds are in ALS patient neural tissues not linked to *SOD1* mutation, suggesting that SOD1 seeding activity may be a promising biomarker, particularly in sporadic ALS cases for whom genetic testing is uninformative.

**Supplementary Information:**

The online version contains supplementary material available at 10.1007/s00401-024-02752-8.

## Introduction

Amyotrophic lateral sclerosis (ALS) is a rapidly progressive neuromuscular disease in which motor neurons degenerate, leaving patients with the inability to innervate skeletal muscle. One in 500 deaths in the United States are because of ALS [59]. Initial symptoms of ALS include fasciculations, muscle fatigue, cramping, and muscle weakness, with more advanced symptoms of ALS including weight loss, loss of speech, and paralysis [36]. Respiratory failure is the most common cause of death for persons with ALS [41]. Approximately, 90% of ALS cases are sporadic (sALS) and the remaining 10% of ALS cases are linked to inherited genetic mutations. The primary inherited genetic mutation in ALS is an abnormal hexanucleotide repeat expansion in the intronic region of Chromosome 9 open reading frame 72 (*C9ORF72*), a gene that encodes a guanine exchange factor to regulate Rab GTPases [20, 25, 47, 56].

Other types of inherited or familial ALS (fALS) mutations include autosomal dominant mutations in the Superoxide Dismutase 1 gene (*SOD1*), which accounts for approximately 20% of fALS cases, or 2% of all ALS [5, 18, 30, 39, 44, 58]. Several autosomal dominant mutations in *SOD1* cause SOD1 to fold abnormally [6], and confer prion-like properties that promote propagation of misfolded SOD1 and neurotoxicity [30, 58]. A new FDA-approved treatment for patients with *SOD1* fALS is now available that reduces expression of SOD1 messenger RNA in patients afflicted with *SOD1* fALS [39]. While SOD1 misfolding and propagation occurs in *SOD*1 fALS and is part of the pathology [12], less is known about wildtype (WT) SOD1 misfolding in other types of ALS, such as sALS [18, 44] and *C9ORF72* fALS [63]. While SOD1 misfolding has been documented in familial ALS linked to mutations in *SOD1* for over 25 years [30, 39], attempts to measure WT misfolded SOD1 in sALS using immunohistochemistry has yielded conflicting results [18, 22, 31, 44, 58, 62, 63]. Thus, alternative methods are needed to increase our understanding of SOD1 dynamics in ALS patients, and to assess the potential utility of SOD1 seeding activity as a biomarker for ALS.

Since the mid-2000s, sensitive seed amplification assays (SAA) such as real-time quaking-induced conversion (RT-QuIC) have been developed to assay pathogenic proteins with self-propagating (i.e., seeding) activity in neurodegenerative diseases [3, 67]. RT-QuIC assays amplify specific amyloid fibrillar, or subfibrillar, protein aggregates in biospecimens based on their ability to recruit recombinant monomers of the same protein (the “substrate”) [3, 28, 32, 60] to the aggregates. The fibrillization kinetics are governed by rates of fibril elongation, fragmentation, and secondary nucleation, (i.e., on the sides rather than the ends of fibrils) and are accelerated by cycles of shaking and rest in a microplate reader. Amplified fibrils are then detected by increased fluorescence intensity of an amyloid binding dye such as Thioflavin T (ThT). RT-QuIC methods have been used to measure multiple disease-specific protein seeds in biospecimens for both fundamental and diagnostic purposes [8, 27, 42]. Diagnostic applications of RT-QuIC for Creutzfeldt–Jakob disease (CJD) [27] and Parkinson’s disease [8, 28, 29] have been proven to be most accurate and impactful to date [29, 43]. With respect to ALS, an RT-QuIC assay for another protein that aggregates in ALS, Transactivation Response DNA Binding protein of 43 kD (TDP-43), has been reported [53]. However, no RT-QuIC SAAs have been reported for ALS-associated forms of SOD1.

SOD1 is a ubiquitously expressed copper/zinc metalloenzyme that converts reactive oxygen species (ROS) to hydrogen peroxide and molecular oxygen [10]. SOD1 may also have other functions connected to the immune system [35] and ROS signaling [66]. In 1993, analysis of ALS patients identified *SOD1* as the first genetic mutation linked to ALS [12]. Numerous mutations in *SOD1* have been identified in *SOD1* fALS patients [1, 59], with some SOD1 mutations such as p.A5V being associated with a more rapid disease course than others [34, 39, 64]. Wildtype SOD1 contains four cysteine (Cys) residues, with two (Cys57 and Cys146) forming a native intramolecular disulfide bond in each monomer, while the other Cys residues have free thiols [51]. These free thiols may contribute to de-stabilization of SOD1 via abnormal cross-linking in ALS linked to mutant *SOD1* [2]. In vitro, the reduced apo form of SOD1 forms soluble oligomers under physiological conditions [7], a process that may have a pathogenic role in ALS in vivo [23]. Immature, structurally disordered SOD1 conformers have also been found to mislocalize and accumulate in spinal cord motor neurons of SOD1-linked and non-SOD1-linked ALS cases, including sALS and *C9ORF72* fALS in a manner specific to regions of neurodegeneration [63]. A conformational ELISA assay has provided evidence that at least one misfolded WT SOD1 conformer is present in cerebrospinal fluid of patients with sALS [61].

Here, we report the development of a SOD1 RT-QuIC assay that detects SOD1 seeding activity in ALS patient postmortem spinal cord and motor cortex tissue. Importantly, this SOD1 RT-QuIC assay detects seeding activity in ALS patients with different etiologies—fALS linked to *SOD1* mutations, fALS linked to abnormal hexanucleotide repeat expansions in *C9ORF72*, and in sporadic ALS patients expressing WT SOD1. We thus provide evidence that the pathology of these distinct types of ALS involves the misfolding of either mutant or WT SOD1 into assemblies with self-propagating (self-seeding) activity. For a subset of sporadic ALS patients examined, SOD1 seeding activity was observed in both primary medial motor cortex and thoracic spinal cord. Analysis of RT-QuIC parameters suggests there may be qualitative differences between the seeds produced in certain subtypes of ALS. Given that these three ALS types comprise ≥ 95% of all ALS cases, SOD1 seeding activity may represent a promising biomarker and therapeutic target.

## Materials and methods

*Preparation of human SOD1 plasmid* cDNA encoding full length WT human SOD1 was manufactured by Genscript (Piscataway NJ, USA) and placed in the pET 28a(+) vector with an N-terminal 6 × His-tag. The human thrombin sequence containing a glycine, serine, serine sequence was removed to prevent any possible cleavage of the 6 × His-tag during protein expression and purification. The cDNA sequence for SOD1 was confirmed by Genscript, and after expression and purification of SOD1, its mass was confirmed by electrospray ionization liquid chromatography mass spectrometry (LC–MS).

*SOD1 expression and purification* The cDNA encoding human SOD1 was expressed from the pET 28(+) vector by transformation into BL21 (New England Biolabs, Ipswich MA, USA; DE3, #C2727I) *E. coli* competent cells using the manufacturer’s protocol. One liter of sterile 2xYT media was inoculated with 10 mL of suspended transformed *E. coli* cells and 1 mL of 50 mg/mL kanamycin and placed in a 2.8 L Fernbach flask. Media was placed in an orbital shaker at 250 rpm, 37 °C, until OD_600_ was between 0.6 and 0.8. At this optical density range, *E. coli* cells were induced with 1 mM final IPTG concentration overnight (~ 15 h) at 30 °C and 150 rpm. Next day, cells were spun for 15 min at 7500 rpm and 4 °C. Cell pellets were used immediately or frozen in − 20 °C. Cells were lysed at a ratio of 50 mL of 0.5 M NaCl, 0.05 M Tris, pH 8.0 (lysis buffer) to 5 g cells, and put on ice with 0.5 mg/mL lysozyme and a stir bar. After mixing on ice, cells were sonicated on ice for 5 min (30 s on/30 s off, 35% AMPL) using a probe sonicator (Sonics VibraCell, Newtown CT, USA). Cell lysate was spun for 30 min at 12,500 rpm and 4 °C. Supernatant was separated carefully from cell pellet using cheesecloth then poured into a 50 mL Falcon tube for metal affinity chromatography. Briefly, a Bio-Rad (Hercules CA, USA) NGC fast protein liquid chromatography system with a 5 mL HisTrap HP column (Cytiva, Marlborough MA, USA) was washed with 0.1 M phosphate, 0.3 M NaCl, 0.02 M imidazole, 2 mM β-ME, pH 7.4 buffer (buffer A), and then 50 mL of cell supernatant was applied to the 5 mL HisTrap HP column, washed with buffer A, then eluted with buffer A in a 0.3 M imidazole (0–100%) gradient (buffer B). We observed two distinct peaks during the imidazole gradient and discovered the later eluting peak with higher imidazole concentration (~ 50% buffer B) was SOD1 (Fig. S1). Eluted SOD1 was exchanged overnight at room temperature in 25 mM Tris pH 8.0 using 10,000 MWCO Snakeskin dialysis tubing. The next day, dialysate was spun down for 30 min, 15,000 rpm at 4 °C. Supernatant was filtered carefully through cheesecloth then concentrated in a 10,000 MWCO Amicon centrifugal concentrator (Millipore Sigma, Burlington MA, USA). Protein purity and identity was assessed with a non-reducing, no heat SDS-PAGE stained with Coomassie (Fig. S1), western blotting (Fig. S2), and electrospray ionization LC–MS (Fig. S3), with expected monomeric SOD1 mass of 17,867.86 Da. Protein concentration was determined using a BCA assay and absorbance at 280 nm with a theoretical extinction coefficient of 5750 M^−1^ cm^−1^ (oxidized) or 5500 M^−1^ cm^−1^ (reduced). The SOD1 concentration via BCA assay and the SOD1 theoretical extinction coefficient from ExPasy were slightly different, with the BCA method producing an experimental extinction coefficient of 5361.5 M^−1^ cm^−1^. We used 5500 M^−1^ cm^−1^ to calculate SOD1 concentration for all experiments.

*Electrospray liquid chromatography native and semi-denaturing mass spectrometry* For semi-denaturing conditions, purified SOD1 was buffer exchanged into 20% acetonitrile with 0.1% formic acid (pH 2.8) using 3 kD molecular weight cutoff filters. For native conditions, SOD1 was buffer exchanged into either 50 mM ammonium acetate with 0.1% acetic acid (pH 4.5) or 100 mM ammonium bicarbonate (pH 8.0). Samples were analyzed using an Agilent 6520 Q-TOF mass spectrometer coupled to an Agilent 1260 UPLC equipped with a AdvancedBio SEC column (300 Å, 2.7 μm, 4.6 × 50 mm). Data were collected in positive ion mode over a range of 500–12,000 *m*/*z*. Gas temperature was 365 °C flowing at 11 L/min with a capillary voltage of 2500 V. For the highest resolution and mass accuracy, reference mass auto correction was enabled (Ref *m*/*z* 922.0098, Agilent). All data were analyzed using Masshunter B.07 with BioConfirm software for protein spectra deconvolution.

*Western blotting* Western blot analysis was performed as described [21]. Briefly, purified SOD1 samples, 10% w/v spinal cord homogenates, or 0.5% (w/v) spinal cord homogenate flow through from immunocapture experiments were run without reducing agent and without heat treatment in Native PAGE buffer (Bio-Rad #1610738), on 12% Bis–Tris Criterion XT gels (Bio-Rad #3450118) in MOPS buffer (Bio-Rad #1610788), transferred to PVDF membrane using a Bio-Rad Trans-Blot Turbo Semi-Dry transfer apparatus (#1704150), and then probed with indicated antibodies. Mouse-anti-SOD1 antibody (BioLegend #850702, clone [O98B10]) was used at 1:2,000 dilution (0.25 µg/mL final). Subsequent incubation with horseradish peroxidase-conjugated donkey-anti-mouse IgG antibodies (Jackson ImmunoResearch #715-035-150) at 1:40,000 dilution (12.5 ng/mL final) was followed by detection with SuperSignal West Pico Plus ECL reagent (ThermoFisher Scientific #34580). ECL signals were detected with an Azure 300 Imager (Azure Biosystems). The acquired images were optimized for image quality with Adobe Photoshop version 24.3 (Adobe).

*Circular dichroism spectroscopy* Purified SOD1 was prepared as described above, except one preparation of SOD1 was purified in the presence of β-ME (reduced, see protein purification methods), and the other SOD1 preparation contained no β-ME. Far-UV secondary structure wavelength scans were performed on 50 µM SOD1 (reduced or non-reduced during purification) in 0.6 M GuHCl, 0.02 M sodium acetate, 0.02 M ThT, pH 4.0 in a 1 cm quartz cuvette with the buffer blank subtracted. Wavelength scans were repeated multiple times, and the mean wavelength scan was used.

*Preparation of human cervical and thoracic spinal cord and motor cortex homogenates* Postmortem human cervical spinal cords, thoracic spinal cords, and primary medial motor cortex with confirmed neuropathological diagnosis of sALS, *SOD1* fALS, and *C9ORF72* fALS with human tissue-matched negative controls (non-neurological and neurological) were received from Neil Cashman and Ian Mackenzie at University of British Columbia, Cindy Ly at Washington University, and the Georgetown Brain Bank. Human spinal cords and motor cortex were weighed to a 10% w/v homogenate using 10 mM HEPES pH 7.4 buffer. For a subset of ALS and negative control cervical cords, solid tissue with buffer were homogenized using a Dounce; for thoracic cords and motor cortex, solid tissue was put in 2 mL screw cap tubes with 1.4 mm ceramic beads (Fisherbrand) and homogenized with a Bead Mill 4 (Fisherbrand) for 60 s on the #4 setting. Spinal cord and motor cortex homogenates were subsequently spun for 5 min at 2000×*g* at 25 °C. Pellets were kept and supernatants were aliquoted in separate 2 mL screw cap tubes, labeled, and frozen at − 80 °C, and subsequently used for RT-QuIC experiments.

*Immunocapture of misfolded SOD1 for SOD1 RT-QuIC assay* Immunocapture was performed with ALS and negative control spinal cord tissue homogenates to control for specificity in the SOD1 RT-QuIC assay. Anti-human SOD1 antibodies O98B10 (pan-SOD1; BioLegend #850702) or C4F6 (misfolded SOD1; MédiMabs #MM-0070-2-P), and isotype-control antibodies mIgG2b (ThermoFisher #MA110427) or mIgG2a (ThermoFisher #MA110418), were cross-linked to Dynabeads M-270 Epoxy (ThermoFisher #14301) according to the manufacturer’s instructions. Beads were isolated with a magnet, washed with PBS, 0.025% (v/v) Tween-20 to remove unbound antibodies and blocked with PBS, 0.1% BSA for 1 h on a tube rotator. Prior to immunocapture, antibody-coated beads were washed once with PBS. For immunocapture, 100 µL beads (3.3 × 10^7^) were incubated with equal volumes (900 µL) of spinal cord homogenates (0.5% w/v in PBS) for 16 h at 4 °C with end-over-end mixing. Flowthrough (unbound fraction) was collected and used in SOD1 RT-QuIC assay and immunoblotting.

*SOD1 RT-QuIC assay* We initially expressed and purified SOD1 from three different *E. coli* strains (T7 #C3013, T7 #2566I, and BL21 (DE3) #C2527I, New England Biolabs) and settled on using BL21 *E. coli* to express all SOD1 substrate for RT-QuIC. For the SOD1 RT-QuIC reaction mix, we examined properties of the SOD1 substrate via mass spectrometry at different pH values, and we observed the apo form of SOD1 below pH 5.0 (Fig. S3). Using buffers below pH 5.0, we initially examined buffers that worked previously using different salts and denaturants with varying concentrations [15, 28, 38]. We altered concentrations of EDTA, GuHCl, β-ME, NaCl, sodium acetate, and substrate concentration, in addition to varying plate reader temperatures and plate reader shake speeds, to discover an optimal condition for a SOD1 RT-QuIC assay not requiring beads. We discovered an optimal reaction mix is 0.6 M GuHCl, 0.02 M sodium acetate, pH 4.0, with a final ThT concentration of 15 µM, with an appropriately prepared human SOD1 substrate concentration of 50 µM (~ 0.9 mg/mL, 5,500 M^−1^ cm^−1^) performed at 500 rpm and 37 °C. SOD1 substrate was used fresh or from aliquots frozen at − 80 °C, and filtered in 100 kD Pall centrifugal filter at 5000×*g*, 15 °C, for 15 min prior to mixing in RT-QuIC buffer. Solutions were made fresh with a 100 mL volumetric flask using ≥ 99% GuHCl (Sigma Aldrich), 99% sodium acetate (Alfa Aesar), and high-performance liquid chromatography grade ddH_2_O (Alfa Aesar). The RT-QuIC reactions were performed in clear bottom 96-well microplates (Thermo-Scientific Nunc 96-well optical bottomed black polystyrene plates w/Lid, catalog #165305). 98 µL of reaction mix (15 µM ThT, 50 µM (~ 0.9 mg/mL) human SOD1 (filtered), 0.6 M GuHCl, 0.02 M sodium acetate, pH 4.0) were put in each well, and seeded with tenfold dilutions of human spinal cord (cervical or thoracic) or motor cortex homogenates diluted in 1 × PBS (made in-house). Plates were sealed with sealing tape (Thermo-Scientific, clear polyolefin, non-sterile, catalog # 232702) and were put in FLUOstar Omega readers (BMG Labtech, Germany) at 37 °C, 500 rpm, with 30 s of shaking and 30 s of resting on a double orbital setting. ThT fluorescence intensity measurements were collected every 40 min with 448 nm excitation and 482 nm emission using a gain setting of 1200. Experiments were conducted using negative and positive control spinal cord and motor cortex homogenate dilutions from 10^–2^ to 10^–5^.

*Transmission electron microscopy of converted and non-converted RT-QuIC products* After experiments ended, the microplate reader was stopped and replicate wells were scraped and pooled, put in screw cap tubes, labeled, and flash frozen in liquid nitrogen and stored at − 80 °C. Converted and non-converted SOD1 RT-QuIC products were negatively stained using 3% aqueous phosphotungstic acid (PTA). Briefly, samples were vortexed and adhered to glow-discharged ultrathin carbon on lacey carbon 400 mesh copper grids (Electron Microscopy Sciences) for 1 min. Samples were lightly blotted, followed by ddH_2_O rinse, lightly blotted, and finally stained with 3% PTA. Grids were imaged in a HT7800 (Hitachi) transmission electron microscope operating at 80 kV. Micrographs were acquired on an XR-81 camera (Advanced Microscopy Techniques).

*RT-QuIC statistical analysis* ThT relative fluorescence units (RFU) versus time were collected with Omega Mars software synced to the microplate readers, then raw data were exported to Microsoft Excel and graphed and analyzed in SigmaPlot 14.5 or 15.0 (Systat software). All RT-QuIC reactions were done in at least quadruplicate for ALS patient tissues and control tissues at 10^–2^, 10^–3^, 10^–4^, and 10^–5^ tissue dilutions. ThT relative fluorescence units (RFU) versus time was plotted for each ALS patient tissue homogenate and each human negative control tissue homogenate at their equal tenfold tissue dilutions. We observed most of our kinetic curves approached stationary phase, but did not entirely plateau, for ALS tissue dilutions. Thus, we fitted raw curves to a model that does not rely on an upper baseline slope to extract RT-QuIC parameters. In Eq. 1,

1 $$y={y}_{0}+\frac{\text{A}}{\left(1+{e}^{-\left(\frac{x-{x}_{0}}{\tau }\right)}\right)},$$

where *y*_0_ is initial ThT fluorescence, *A* is ThT amplitude (final – initial fluorescence), *x*_0_ is time to reach 50% ThT fluorescence, and *τ* is a fibril time constant. The time to fluorescence positivity threshold or lag phase is equal to *x*_0_ – 2*τ* [16]. We fit our raw data to this model for each kinetic curve using constraints > 0, to estimate *x*_0,_ *y*_0_, *τ*, and *A*. When *x* equals *x*_0_, Eq. 1 reduces to Eq. 2:

2 $${y}_{50}={y}_{0}+\frac{A}{2}.$$

We used *y*_0_ and *A* from fits to determine *y*_50_ values (i.e., 50% ThT fluorescence), and used *τ* and *x*_0_ from fits to calculate lag phase (*x*_0_ − 2*τ*). We plotted mean 50% ThT fluorescence (RFU) versus lag phase (hours) to examine the correlation between these two variables. After graphing 50% ThT fluorescence versus lag phase for each ALS patient neural tissue at 10^–3^, 10^–4^, and 10^–5^ dilutions, we used Eq. 3 to examine the dependence of 50% ThT fluorescence on lag phase, and determine the linear correlation coefficient R. In Eq. 3,

3 $${y}_{50}= \beta +m\times \alpha ,$$

where *β* is *y*_50_-intercept, m is slope, and α is lag phase. To determine the accuracy of this novel SOD1 RT-QuIC assay applied to patient neural tissues, we determined sensitivity and specificity using receiver operating characteristic (ROC) plots [42]. ALS and non-neurological and neurological spinal cord kinetic data were examined at 10^–3^ (threshold equal to 5000 RFU and 125 h) and 10^–4^ (threshold equal to 5000 RFU and 175 h) dilutions to determine sensitivity and specificity and area under the ROC curves. Sensitivity (*y*-axis) vs. 1 − Specificity (*x*-axis) was plotted at each dilution using ROC analysis from GraphPad Prism 10.2.2.

## Results

### Properties of human WT SOD1 substrate for RT-QuIC

RT-QuIC assays require non-fibrillar substrate protein molecules that can be recruited into growing fibrils in the presence of pre-existing ex vivo seeds more rapidly than they spontaneously nucleate into seeding-competent assemblies under assay conditions. Such non-fibrillar substrates are not necessarily identical to the physiological forms of the given protein and its thermodynamic stability. Since native WT SOD1 contains two free cysteine thiols and one intramolecular disulfide bond per monomeric unit [51], histidine-tagged WT human SOD1 (recombinant SOD1; rSOD1) was grown in BL21 *E. coli* and purified in the presence or absence of the reducing agent β-ME using metal-ion affinity chromatography. Non-reducing SDS-PAGE gels of eluted fractions exposed to SDS without heating indicated the presence of rSOD1 monomers and dimers in a major peak (pk2) of eluted protein (Fig. S1). After dialysis, native PAGE gels, and immunoblotting with anti-SOD1 antibodies indicated that in both a Tris buffer and RT-QuIC reaction buffer, rSOD1 was primarily monomeric (Fig. S2). The mass of rSOD1 prepared with or without β-ME was confirmed, with the presence of β-ME allowing formation of an intramolecular disulfide bond (Fig. S3) [19]. This disulfide was not present in preparation of rSOD1 without β-ME (Fig. S3), suggesting *E. coli* did not form the disulfide bond in rSOD1. Using electrospray ionization LC–MS, we examined metal occupancy of rSOD1 at different pHs. This analysis indicated the apoenzyme predominated at low pH (pH 2.8) while copper was likely bound at higher pH (pH 5.5) (Fig. S3). Circular dichroism spectroscopy in the far-UV region showed rSOD1 prepared with or without β-ME differed in secondary structure (Fig. S4). Reduced ellipticity at 230 nm suggested a loss of polyproline II helix secondary structure [9, 55] in the β-ME-treated rSOD1 in RT-QuIC buffer where the CD spectrum was random coil; this was not observed with rSOD1 in RT-QuIC buffer prepared without β-ME treatment. In summary, these data provided evidence that β-ME-treated rSOD1 substrate used in RT-QuIC assays below was largely monomeric (Fig. S2) with one intramolecular disulfide bond (Fig. S3), and was random coil (Fig. S4) and metal-free (Fig. S3).

In developing SAAs, it can be difficult to predict optimal characteristics of the substrate protein because it must simultaneously be stable enough to limit spontaneous nucleation and fibrillization yet be de-stabilized enough to refold in the presence of preformed ex vivo seeds. Since the melting temperature (*T*_m_) of rSOD1 is drastically reduced in its apo form (*T*_m_ of ~ 42 °C versus 85 °C in the metal-bound form) [64], we reasoned a temperature in the 42 °C range may be suitable for RT-QuIC. Importantly, and somewhat paradoxically, the reductant (β-ME)-treated rSOD1 substrate, which was buffer exchanged to form a single intramolecular disulfide bond (Fig. S3), was an efficient substrate for RT-QuIC. The non-reduced rSOD1 substrate failed to give a ThT-positive product, suggesting BL21 *E. coli* were not able to form this intramolecular disulfide bond in rSOD1. Perhaps rSOD1 was initially trapped in a state, e.g., an intermolecularly disulfide-linked form, which precluded the required intramolecular disulfide bond formation without transient β-ME treatment. For example, the β-ME might have reduced intermolecular disulfide crosslinks formed in *E. coli*, allowing monomers access to the intramolecularly disulfide-linked monomeric form upon removal of the β-ME. Indeed, native PAGE and Western blotting showed rSOD1 substrate in RT-QuIC buffer was primarily monomeric compared to the dimer-monomer mixture seen prior to β-ME treatment and buffer exchange (Fig. S2).

### Seeding of rSOD1 fibrillization by ALS spinal cord homogenates in RT-QuIC conditions

**Fig. 1:**
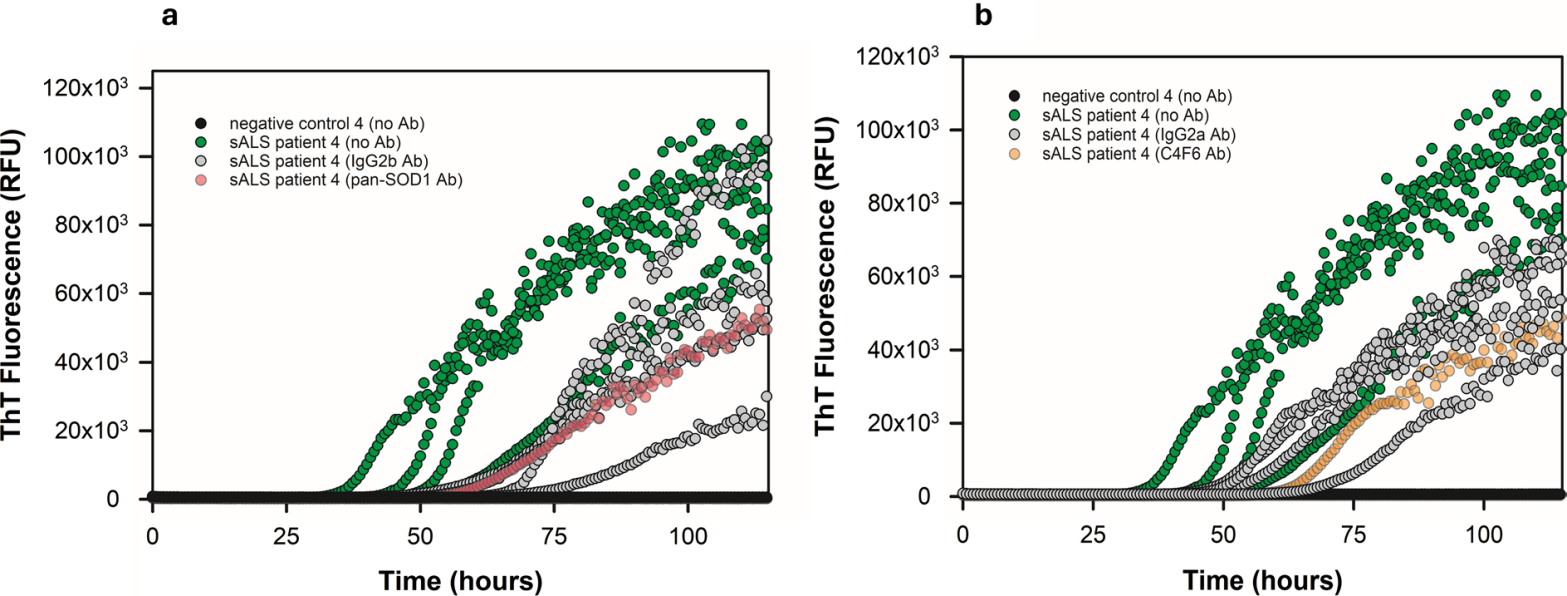
Immunodepletion of SOD1 seeding activity using anti-SOD1 antibodies. **a** SOD1 RT-QuIC of supernatants of sALS spinal cord homogenate exposed to beads alone (green), or beads linked to either a pan-SOD1 antibody (pink) or an isotype-control antibody (gray). **b** Same as panel **a**, except for the use of the C4F6 SOD1 antibody (orange) and a different isotype-control antibody treatment (gray). A non-ALS negative control spinal cord homogenate was included without antibody treatment and remained ThT-negative throughout. All samples at 5 × 10^–3^ spinal cord tissue dilutions. ThT fluorescence traces from individual quadruplicate wells are shown

Given SOD1 pathology is commonly observed in spinal cord of *SOD1* fALS patients, we sought initial confirmation of the presence of abnormal SOD1 in spinal cord homogenates from six ALS cases and two human negative controls by western blotting using a pan-SOD1 antibody. Although the control spinal cords gave a broad smear of bands above the size of the SOD1 monomer, spinal cords from patients with *SOD1* fALS, sALS, or *C9ORF72* fALS, tended to have enhanced intensities and distinct patterns of larger bands suggestive of greater abundance of higher order structures (Fig. S5). Using a SOD1 RT-QuIC assay, we were able to discern clear kinetic discrimination of SOD1 seeding activity between ALS patient cord homogenates and negative control cord homogenates. For example, seeding with a 5 × 10^–3^ dilution of a sALS spinal cord homogenate gave enhanced ThT fluorescence in ~ 32–55 h, while controls remained negative for > 100 h (Fig. 1). These results provided initial evidence of sALS-associated SOD1 RT-QuIC seeding activity.

### Immunodepletion of sALS-associated seeding activity with anti-SOD1 antibodies

We then investigated the SOD1 specificity of our assay by performing immunodepletion experiments. A sALS cord homogenate was either left untreated or incubated with magnetic beads cross-linked to the following antibodies: an antibody to misfolded SOD1 (C4F6), a pan-SOD1 antibody, or two isotype-matched control antibodies. The beads were removed, and the remaining spinal cord supernatants assayed by SOD1 RT-QuIC. We observed a greater reduction in SOD1 seeding activity with SOD1 antibodies (C4F6 and a pan-SOD1 antibody) than with isotype-matched control antibodies (Fig. 1), providing evidence that at least some of the seeding activity in the sALS spinal cord contained SOD1 (Table 1). Immunoprecipitation experiments with the sALS spinal cord homogenate showed a greater reduction of SOD1 protein when captured with SOD1 specific antibodies relative to capture using isotype-control antibodies (Fig. S10).

**Table 1:** SOD1 RT-QuIC parameters for immunodepletion of sALS spinal cord homogenate

Sample	Lag phase (h)	50% ThT (RFU)
sALS patient 4 (no Ab)	48.37 ± 11.03	43,500 ± 5467
sALS patient 4 (pan-SOD1 Ab)	62.68*	27,293*
sALS patient 4 (pan-SOD1 isotype-control Ab)	70.22 ± 6.803^	32,100 ± 19,029^
sALS patient 4 (C4F6 Ab)	62.41*	21,878*
sALS patient 4 (C4F6 isotype-control antibody)	57.56 ± 10.35^	27,600 ± 6406^

*1/4 wells ThT positive

^3/4 wells ThT negative

### SOD1 seeding activity in *SOD1* fALS, *C9ORF72* fALS, and sporadic ALS patient spinal cord

**Fig. 2:**
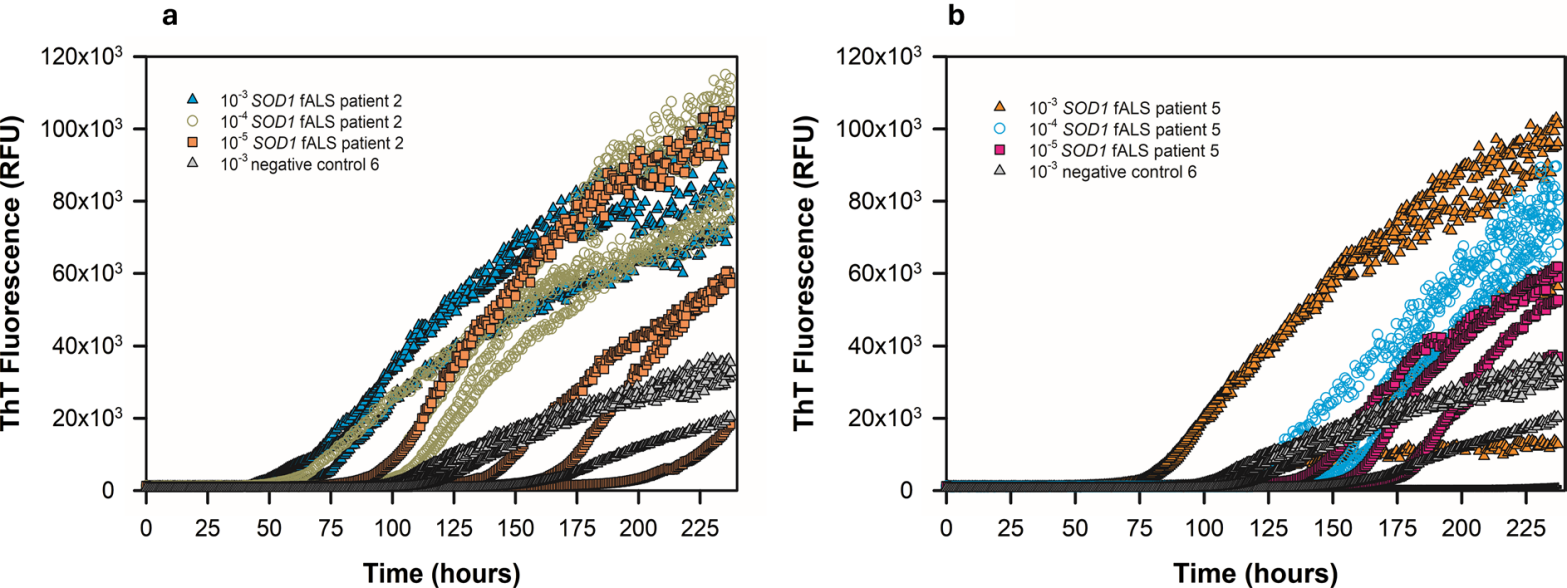
SOD1 RT-QuIC plot of cervical spinal cord homogenates from *SOD1* familial ALS patients 2 (**a**) and 5 (**b**) at 10^–3^—10^–5^ dilutions. Data from non-ALS control cervical spinal cord (10^–3^ dilution) shown

**Fig. 3:**
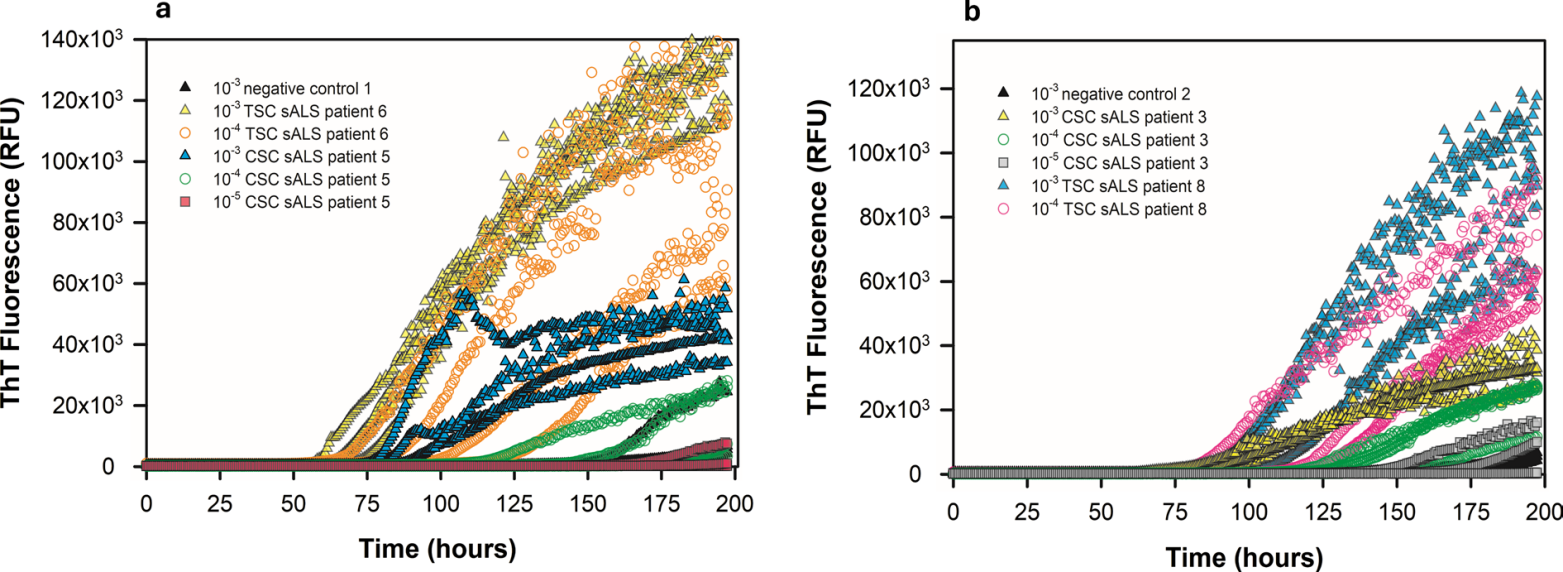
SOD1 RT-QuIC plot of sporadic ALS cases using cervical and thoracic spinal cord homogenates. In **a**, sporadic ALS patients 5 (cervical) and 6 (thoracic) are shown, and in **b**, sporadic ALS patients 3 (cervical) and 8 (thoracic) are shown at the designated dilutions. Data from non-ALS control spinal cords are shown for comparison

**Fig. 4:**
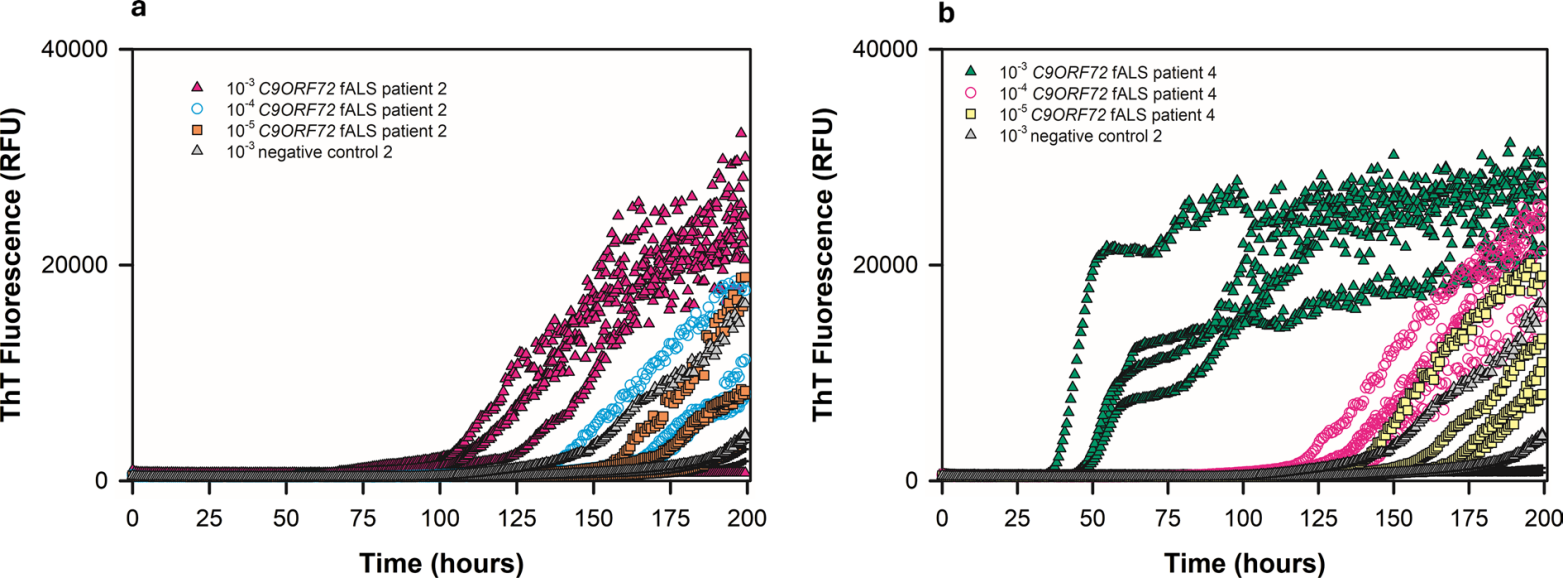
SOD1 RT-QuIC plot of cervical spinal cord homogenates from *C9ORF72* fALS patients 2 (**a**) and 4 (**b**) compared to non-ALS control spinal cord at the designated dilutions

**Fig. 5:**
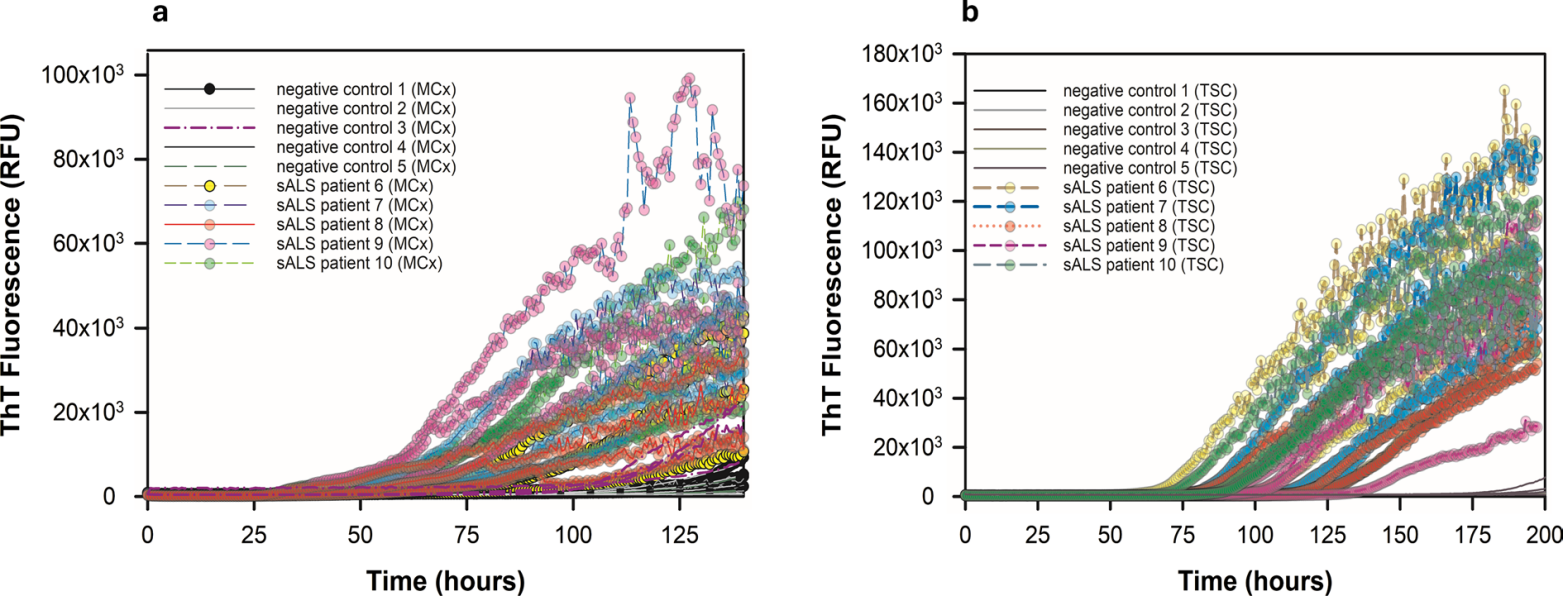
sALS patients 6, 7, 8, 9, 10 motor cortex (**a**) and thoracic cord (**b**) versus tissue-matched controls at 10^–4^ dilution

Next, we expanded our study by comparing spinal cord tissue from patients clinically diagnosed with sALS (*n* = 10), fALS linked to mutations in *SOD1* (*n* = 5) or *C9ORF72* (*n* = 5), or human negative controls (*n* = 12). For 15 of the 20 total ALS patients, cervical spinal cord was used. For 5 sALS patients, thoracic spinal cord specimens were analyzed. We homogenized spinal cords at 10% w/v and assayed tenfold serial dilutions thereof. At 10^–2^, we observed matrix inhibition of ThT fluorescence in several ALS spinal cord homogenates, but at 10^–3^, 10^–4^, and 10^–5^ tissue dilutions, the sALS, *SOD1* fALS, and *C9ORF72* fALS spinal cords usually gave enhanced ThT fluorescence compared to that elicited by the negative control spinal cords (Figs. 2, 3, 4, 5). The sALS and *C9ORF72*-linked fALS cervical cord specimens gave substantially lower ThT fluorescence intensity relative to those elicited by *SOD1* fALS cervical and sALS thoracic cords at same dilutions. Collectively, these data provide evidence that SOD1 RT-QuIC can detect SOD1 seeds in neural tissue of patients with sporadic and genetic etiologies of ALS.

### SOD1 seeding activity in thoracic cord and motor cortex of sporadic ALS patients

Since ALS pathology can have neurodegeneration of upper and lower motor neurons [14, 59], we further examined medial primary motor cortex of five sporadic ALS patients and compared these seeding activity results to their thoracic cord (Table 2, sALS patients 6, 7, 8, 9, and 10) and to eight tissue-matched non-ALS negative controls (neurological and non-neurological, Table 2). Medial primary motor cortex was homogenized at 10% w/v and run in SOD1 RT-QuIC by seeding wells with brain dilutions from 10^–2^ to 10^–5^. At 10^–2^ and 10^–3^, we observed no ThT fluorescence, probably because of motor cortex matrix inhibition of ThT; however, at 10^–4^ and 10^–5^ tissue dilutions, we observed substantial ThT fluorescence in all five sporadic ALS patient’s motor cortex relative to non-ALS motor cortex controls (Fig. 5a). The SOD1 seeding activity comparison between these two anatomical regions in each sporadic ALS patient suggests their motor cortex lag phase is shorter than their thoracic cord (Fig. 7**)**.

**Table 2:** Non-ALS and ALS patient information

non-ALS patients	Neural tissue	Diagnosis	Age	Gender	Disease duration	Pathology
Negative 1	TSC, MCx	–	67	Male	–	Mild patchy ischemic changes, moderate patchy atherosclerosis
Negative 2	TSC, MCx	–	71	Male	–	Mild hypoxic ischemic changes
Negative 3	TSC, MCx	–	70	Male	–	Moderate hypertensive vasculopathy
Negative 4	TSC, MCx	–	75	Male	–	Focal hypoxic ischemic changes
Negative 5	TSC, MCx	–	71	Male	–	Mild acute hypoxic encephalopathy, moderate atherosclerosis
Negative 6	CSC	–	–	–	–	–
Negative 7	CSC	–	–	–	–	–
Negative 8	CSC, TSC, MCx	CBD	84	Female	–	Corticobasal degeneration (cortical pTau inclusions)
Negative 9	CSC, TSC, MCx	Clinical FTD	65	Male	–	N/A
Negative 10	CSC, TSC, MCx	AD/DLBD	73	Male	–	AD/DLBD
Negative 11	CSC	Alcoholic cirrhosis	46	Male	–	Cerebral atrophy
Negative 12	CSC	Respiratory failure	81	Male	–	LM fibrosis of spinal cord, no ALS pathological change

ALS patients	Neural tissue	Diagnosis	Age	Gender	Disease duration (months)	Neuropathology
*SOD1* fALS patient 1	CSC	SOD1 ALS	67	Female	24	ALS-SOD
*SOD1* fALS patient 2	CSC	SOD1 ALS	76	Female	6	ALS-SOD
*SOD1* fALS patient 3	CSC	SOD1 ALS	46	Male	36	ALS-SOD
*SOD1* fALS patient 4	CSC	SOD1 ALS	67	Male	120	ALS-SOD
*SOD1* fALS patient 5	CSC	SOD1 ALS	63	Male	192	ALS-SOD
sALS patient 1	CSC	sALS	57	Male	12	ALS-TDP, FTLD-TDP
sALS patient 2	CSC	sALS	65	Female	24	ALS-TDP
sALS patient 3	CSC	sALS	67	Male	24	ALS-TDP, senile plaques (CERAD moderate)
sALS patient 4	CSC	sALS	75	Male	12	ALS-TDP, AD diffuse LBD
sALS patient 5	CSC	sALS	51	Male	24	ALS-TDP, FTLD-TDP
sALS patient 6	TSC, MCx	sALS	81	Male	13	ALS w/ TDP-43 inclusions in motor cortex and lumbar spinal cord, PART
sALS patient 7	TSC, MCx	sALS	69	Male	14	ALS w/TDP-43 inclusions in motor cortex
sALS patient 8	TSC, MCx	sALS	79	Female	19.5	ALS
sALS patient 9	TSC, MCx	sALS	42	Male	22.5	ALS w/TDP-43 inclusions
sALS patient 10	TSC, MCx	sALS	64	Female	26.6	ALS w/TDP-43 inclusions in brain stem and spinal cord
*C9ORF72* fALS patient 1	CSC	C9 ALS	51	Male	24	ALS-TDP, DPR pathology
*C9ORF72* fALS patient 2	CSC	C9 ALS	59	Female	12	ALS-TDP, DPR pathology, tauopathy
*C9ORF72* fALS patient 3	CSC	C9 ALS	51	Male	12	ALS-TDP, DPR pathology
*C9ORF72* fALS patient 4	CSC	C9 ALS	70	Female	24	ALS-TDP, DPR pathology, FTLD-TDP
*C9ORF72* fALS patient 5	CSC	C9 ALS	58	Female	24	ALS-TDP, DPR pathology, FTLD-TDP

*CSC* cervical spinal cord, *TSC* thoracic spinal cord, *MCx* motor cortex, *CBD* corticobasal degeneration, *FTD* frontotemporal dementia, *AD/DLBD* Alzheimer’s disease and dementia with Lewy body disease, *ALS-SOD* ALS with *SOD1* mutation, *ALS* amyotrophic lateral sclerosis, *sALS* sporadic ALS, *C9* *ALS* ALS with *C9ORF72* mutation, *ALS-TDP* ALS with transactivation response DNA binding protein of 43 kD (TDP-43) inclusions, *FTLD-TDP* frontotemporal lobar degeneration with TDP-43 inclusions, *CERAD* Consortium to Establish a Registry for Alzheimer’s disease neuropathology protocol for dementia with Lewy bodies, *LBD* Lewy Body dementia, *DPR* dipeptide repeat pathology

### Electron microscopy of SOD1 RT-QuIC products

ThT positivity of amplified ALS-seeded SOD1 RT-QuIC kinetic curves suggested they are amyloid fibrils. To confirm this visually, we analyzed the ThT-positive products of SOD1 RT-QuIC reactions seeded with sALS and fALS spinal cord homogenates by negative stain transmission electron microscopy and found abundant fibrils (Fig. S6). Fibrils were also observed in reactions initiated with negative control cord homogenates when the latter were allowed to incubate long enough to become ThT-positive, presumably because of eventual spontaneous nucleation of rSOD1 fibrillization.

### Analysis of SOD1 RT-QuIC parameters

**Fig. 6:**
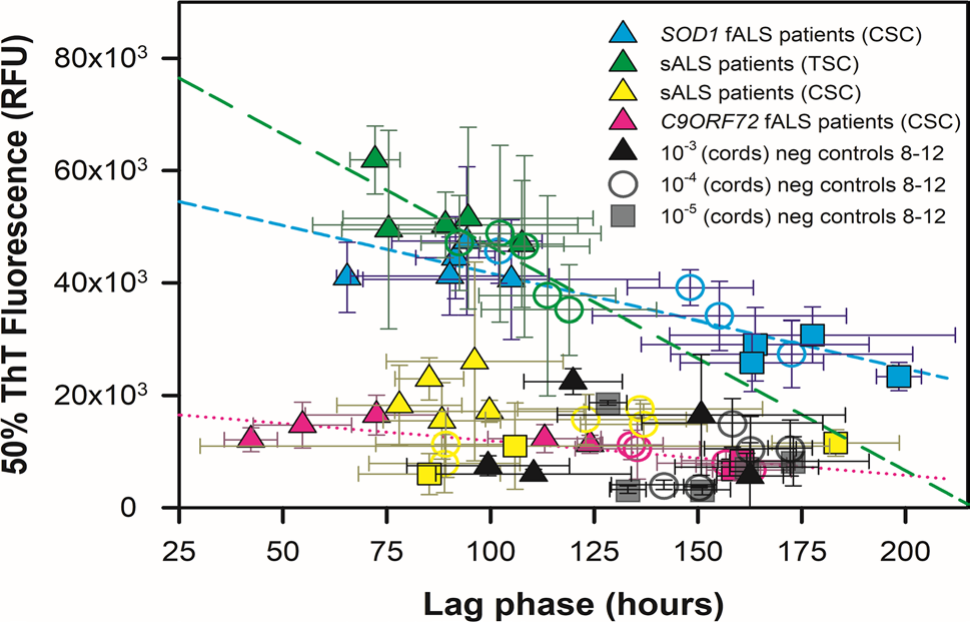
50% ThT fluorescence (RFU) versus lag phase (hours) for ALS patient spinal cord homogenates and neurological negative control spinal cords at 10^–3^ (triangles), 10^–4^ (circles), and 10^–5^ (rectangles) dilutions. Colors represent ALS type. Dashed lines are fits to Eq. 3 for each ALS type, except sALS cervical cords

**Fig. 7:**
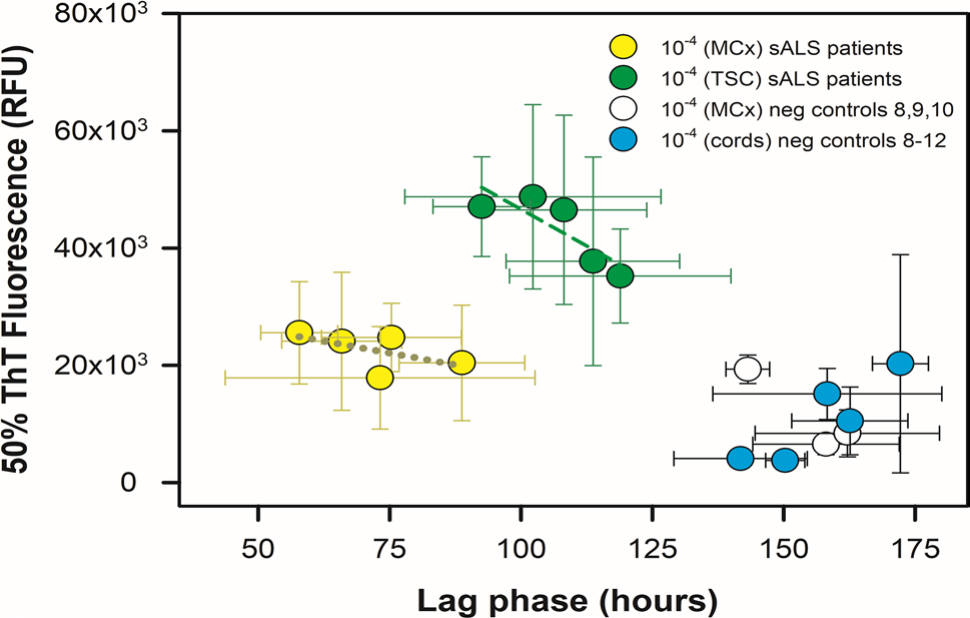
50% ThT fluorescence (RFU) versus lag phase (hours) for motor cortex dilutions (yellow circles) and thoracic spinal cord dilutions (green circles) at 10^–4^ for sALS patients 6, 7, 8, 9, 10 with neurological negative controls 8, 9, 10, 11, 12 (white and blue)

**Fig. 8:**
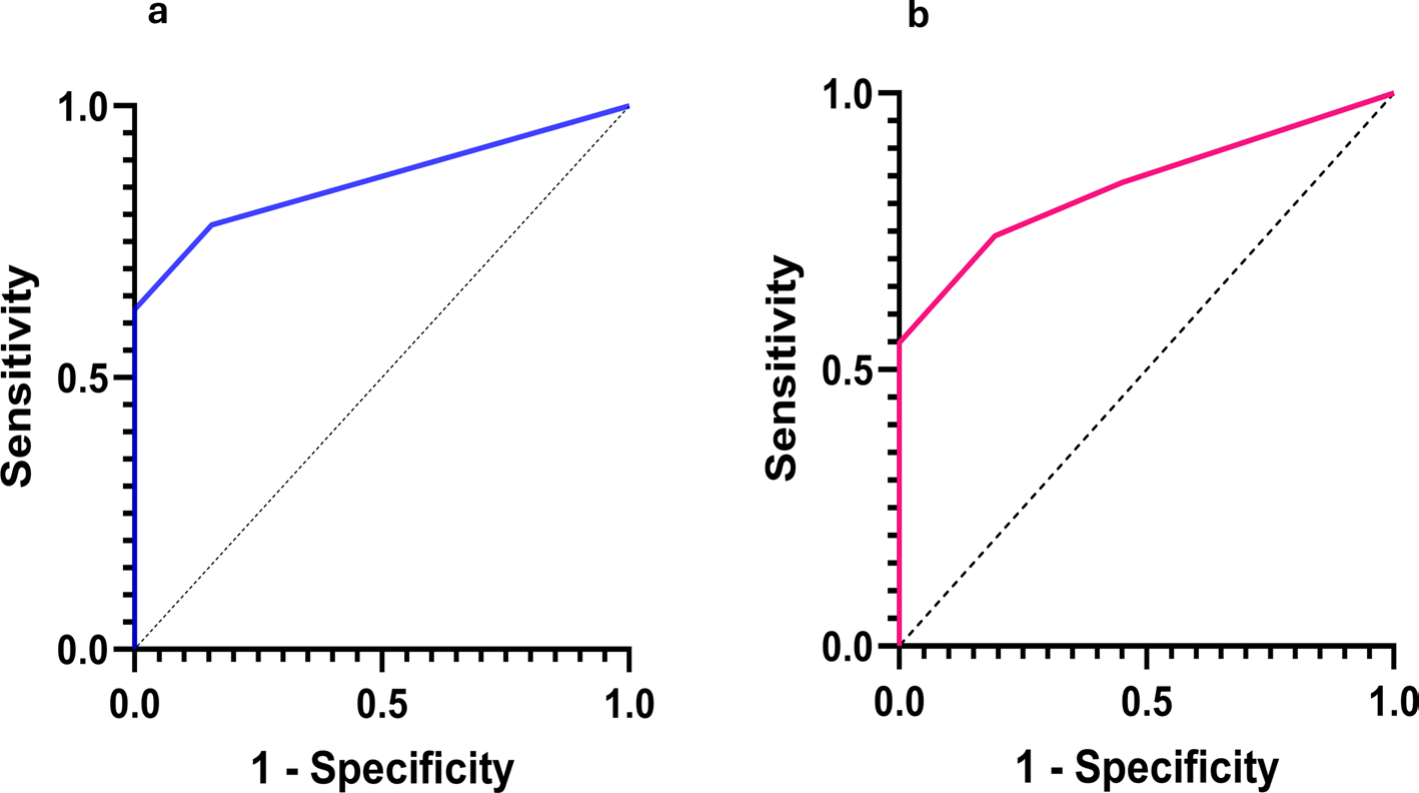
Receiver operating characteristic curves for SOD1 RT-QuIC assay at 10^–3^ spinal cord dilutions (threshold 5000 RFU and 125 h) (**a**) and 10^–4^ spinal cord dilutions (threshold 5000 RFU and 175 h) (**b**)

When SOD1 RT-QuIC experiments ended, we fit each kinetic curve to Eq. 1 to extract its ThT fluorescence amplitude, time to 50% ThT fluorescence, initial ThT fluorescence, and a fibril time constant (Figs. 6, 7). Figure S8 shows examples of fits to the kinetic data. From the fits from Eq. 1, we used these four parameters to calculate 50% ThT fluorescence and lag phase (time to a designated threshold of positive fluorescence), as previously described [16]. For each ALS patient spinal cord and motor cortex dilution, we calculated mea* n* ± standard deviation for lag phase and 50% ThT fluorescence, and graphed 50% ThT fluorescence versus lag phase for each ALS neural tissue dilution. In Fig. 6, we observed in general that 50% ThT fluorescence decreased, and lag phase extended, as ALS spinal cords were diluted from 10^–3^ to 10^–5^. For each ALS dataset type (i.e., cervical cords from *SOD1* fALS patients, thoracic cords from sALS patients, cervical cords from sALS patients, cervical cords from *C9ORF72* fALS patients), we used Eq. 3 to examine correlation between 50% ThT vs. lag phase. This analysis yielded high linear correlation coefficients for *SOD1* fALS cervical cords (*R* = 0.88), *C9ORF72* fALS cervical cords (*R* = 0.85), and sporadic ALS thoracic spinal cords (*R* = 0.84). For motor cortices from sporadic ALS patients, we observed a similar relationship between 50% ThT vs. lag phase (Fig. 7**,** *R* = 0.57). Thus, lag phase and 50% ThT fluorescence were strongly negatively correlated in most of these ALS patient neural tissues, which likely was dependent on SOD1 seed concentration (Table 3 and Figs. 6, 7). Most non-neurological ALS control spinal cords at these dilutions failed to elicit ThT fluorescence until ~ 175 h at 10^–3^ dilution and ~ 200 h for 10^–4^ and 10^–5^ cord dilutions (Fig. S7). Negative control 6 (Fig. S9) gave positive ThT fluorescence at ~ 125 h at 10^–3^ dilution, while at 10^–4^ and 10^–5^ dilutions, negative controls 6 and 7 gave ThT positivity at ~ 160 h and > 200 h respectively. To better understand the specificity of our SOD1 RT-QuIC assay, we examined neurological tissue-matched specimens from patients clinically diagnosed with other neurological diseases (#s 8–12; Figs. 7, S11–S19; Table 2). The mean ThT fluorescence responses induced by these controls were slower and weaker than those elicited by tissue-matched ALS specimen. We evaluated the accuracy of our SOD1 RT-QuIC assay by establishing a threshold (see methods) to determine sensitivity and specificity using receiver operating characteristic curves or ROC plots [42]. Figure 8 shows two ROC plots for all cervical and thoracic spinal cord data at 10^–3^ and 10^–4^ dilutions, and, using highest likelihood ratios, sensitivity, specificity, and area under ROC curve are 0.688, 0.938, and 0.861 for 10^–3^ dilutions, and 0.742, 0.806, and 0.833 for 10^–4^ dilutions, respectively (Table 4).

**Table 3:** SOD1 RT-QuIC parameters (mean ± standard deviation) on patient neural tissue

	Lag phase (h)			50% ThT fluorescence (RFU)		
	10^–3^	10^–4^	10^–5^	10^–3^	10^–4^	10^–5^
*ALS patient*						
*SOD1* fALS patient 1^^^	91.5 ± 9	172 ± 29	–	44,463 ± 7307	27,348 ± 5979	–
*SOD1* fALS patient 2^^^	64.6 ± 2	76.5 ± 51	177 ± 34	40,939 ± 6273	45,659 ± 5748	30,000 ± 4962
*SOD1* fALS patient 3^^^	90.2 ± 24	155 ± 30	163 ± 27	41,200 ± 6964	34,100 ± 6131	29,100 ± 6502
*SOD1* fALS patient 4^^^	94.2 ± 18	202 ± 18	196 ± 5	47,439 ± 13,233	26,523 ± 8298	17,521 ± 11,860
*SOD1* fALS patient 5^^^	104 ± 35	148 ± 15	163 ± 17	40,600 ± 10,623	39,100 ± 3193	25,809 ± 5144
sALS patient 1^^^	99.7 ± 22	122 ± 24	183 ± 15	17,161 ± 1958	15,708 ± 4449	11,533 ± 2373
sALS patient 2^^^	78 ± 15	89 ± 18	85 ± 17	18,215 ± 7152	7854 ± 5189	5997 ± 3661
sALS patient 3^^^	96 ± 21	136 ± 15	–	26,024 ± 17,685	14,834 ± 3631	–
sALS patient 4^^^	88 ± 14	89 ± 16	105 ± 75	15,472 ± 6820	11,269 ± 5886	10,974 ± 7683
sALS patient 5^^^	85 ± 8	136 ± 29	–	22,948 ± 3751	17,594 ± 1571	–
sALS patient 6*	72.1 ± 6	102 ± 24	–	61,900 ± 6053	48,800 ± 15,707	–
sALS patient 6^#^	–	88.7 ± 12	112 ± 0.67	–	20,400 ± 9856	13,672 ± 797
sALS patient 7*	89 ± 31	108 ± 15	–	50,300 ± 5835	46,500 ± 16,140	–
sALS patient 7^#^	–	65.8 ± 11	101 ± 7.2	–	24,100 ± 11,787	19,793 ± 4916
sALS patient 8*	107 ± 10	118 ± 21	–	46,900 ± 11,332	35,200 ± 8046	–
sALS patient 8^#^	–	75.3 ± 13	84.5 ± 8.1	–	24,700 ± 5816	22,370 ± 5226
sALS patient 9*	75 ± 11	113 ± 16	–	49,500 ± 17,642	37,700 ± 17,796	–
sALS patient 9^#^	–	73.2 ± 29	104.5 ± 26	–	17,852 ± 8750	13,956 ± 4311
sALS patient 10*	94 ± 30	92 ± 9	–	51,500 ± 16,218	47,100 ± 8480	–
sALS patient 10^#^	–	57.8 ± 7	134.4 ± 67	–	25,500 ± 8735	18,596 ± 13,708
*C9ORF72* fALS patient 1^^^	123 ± 3	163 ± 9	158 ± 6.4	11,300 ± 1621	6710 ± 1377	6720 ± 1468
*C9ORF72* fALS patient 2^^^	113 ± 13	156 ± 16	–	12,300 ± 2496	7940 ± 3015	–
*C9ORF72* fALS patient 3^^^	72.5 ± 17	135 ± 15	160 ± 10	16,500 ± 3,545	10,500 ± 5432	7610 ± 2820
*C9ORF72* fALS patient 4^^^	42.2 ± 6	133 ± 7	160 ± 14	12,100 ± 2127	11,200 ± 2614	8380 ± 1268
*C9ORF72* fALS patient 5^^^	54.6 ± 11	–	–	14,700 ± 4086	–	–
*Non-ALS patient*						
Negative control 6^^^	130 ± 10	159 ± 42	194 ± 46	49,400 ± 9833	39,700 ± 15,683	23,700 ± 17,605
Negative control 7^^^	170 ± 16	203 ± 22	–	49,800 ± 14,864	30,000 ± 5995	–
Negative control 8^^^	110 ± 23	141 ± 12	152 ± 7	6000 ± 820	4082 ± 852	3120 ± 1071
Negative control 8^#^	148 ± 19	158 ± 13	164 ± 16	4076 ± 2942	6520 ± 1763	7140 ± 1197
Negative control 9^*^	150 ± 34	192 ± 41	172 ± 18	41,400 ± 37,744	69,600 ± 85,742	14,100 ± 2314
Negative control 9^#^	144 ± 16	162 ± 17	156 ± 3.2	8254 ± 2851	8390 ± 3996	5216 ± 947
Negative control 10^*^	176 ± 17	172 ± 5	161 ± 17	15,000 ± 17,166	20,278 ± 18,623	12,862 ± 13,602
Negative control 10^#^	106 ± 23	143 ± 4	144 ± 20	20,568 ± 694	19,314 ± 2,415	17,379 ± 3,750
Negative control 11^^^	99 ± 19	150 ± 3	133 ± 4	7464 ± 1785	3770 ± 226	3215 ± 665
Negative control 12^	120 ± 12	158 ± 21	128 ± 5	22,474 ± 2299	15,113 ± 4335	18,699 ± 405

^^^CSC

*TSC

^#^MCx

**Table 4:** ROC curve parameters for 20 ALS and 12 non-ALS spinal cords

10^–3^ spinal cord dilutions							
Area under ROC curve			Sensitivity	95% CI	Specificity	95% CI	Likelihood ratio
Area	0.861	> 0.125	0.781	0.612–0.890	0.844	0.682–0.931	5.0
Std error	0.0493	> 0.375	0.688	0.514–0.820	0.938	0.799–0.989	11.0
95% CI	0.765–0.958	> 0.75	0.625	0.453–0.771	1.00	0.893–1.0	–
*p* value	< 0.0001						

10^–4^ spinal cord dilutions							
Area under ROC curve			Sensitivity	95% CI	Specificity	95% CI	Likelihood ratio
Area	0.833	> 0.125	0.839	0.674–0.929	0.548	0.378–0.708	1.86
Std error	0.0536	> 0.375	0.742	0.568–0.863	0.806	0.637–0.908	3.83
95% CI	0.728–0.938	> 0.625	0.548	0.378–0.708	1.00	0.89–1.0	–
*p* value	< 0.0001	> 0.875	0.387	0.237–0.562	1.00	0.89–1.0	–

## Discussion

The need to identify new biomarkers for ALS has never been greater, given the advent of novel disease-modifying treatments. The time from symptom onset to diagnosis can be delayed from ~ 10 to 16 months for ALS patients in current healthcare settings [48]. Diagnostic delay can be influenced by poor recognition of ALS among general practitioners because of disease rarity as well as heterogeneity in phenotypes and progression rates. Clinical criteria for definite ALS are rarely met at initial presentation. Lack of recognition has resulted in some patients undergoing unnecessary diagnostic testing, misdiagnosis, and delayed initiation of appropriate therapies [49]. The greatest diagnostic delay is in limb-onset patients because they are frequently misdiagnosed with degenerative spine disease or peripheral neuropathies [48]. Here, we report proof-of-concept that a novel SOD1 seed amplification assay can detect misfolded SOD1 in patient spinal cords and motor cortices with familial (*SOD1, C9ORF72*) or sporadic cases of ALS (i.e., ≥ 90% of all ALS cases), which may translate to a potential biomarker for this neuromuscular disease. Some applications of a SOD1 seeding activity biomarker could be to screen biofluids from patients who do not have a mutation in *SOD1*, and to determine variants in patients with unknown pathogenicity. As has been demonstrated with RT-QuIC assays for other pathological protein assemblies [43, 52], our prototypic SOD1 RT-QuIC assay might be adaptable to detection of SOD1 seeds in clinically accessible biospecimens for these purposes. However, the collective experience in developing prior RT-QuIC assays suggests sample processing and assay conditions may have to be modified for the analysis of new types of biospecimens.

### ALS-associated SOD1 seeding activity in sALS, *SOD1* fALS, and *C9ORF72* fALS spinal cords

The development of a SOD1 RT-QuIC seed amplification assay allows us to address key issues that relate to the involvement of SOD1 misfolding in ALS. Our detection of SOD1 seeding activity in spinal cords and motor cortices of patients with sALS, *SOD1* fALS, and *C9ORF72* fALS provides evidence that in each ALS type, misfolded SOD1 has prion-like seeding, or self-propagating activity, which might underpin the commonly observed spreading of pathology from localized anatomical sites to other sites within a patient over time. This observation is particularly notable in cases of sALS and *C9ORF72* fALS, where the involvement of WT SOD1 misfolding has historically been less clear [18, 22, 24, 31, 34, 62, 63, 65]. To our knowledge, the detection of SOD1 seeding activity via cell-free seed amplification (i.e., RT-QuIC) analysis of neural tissues from ALS patients is novel. From a practical perspective, our SOD1 RT-QuIC assay provides a sensitive and specific method for detecting abnormal SOD1 (WT and mutant), that is orthogonal to apparently less sensitive methods such as ELISA or Western blotting. As such, SOD1 RT-QuIC should be a new tool for measuring SOD1 misfolding in pathogenesis studies and in the development of therapeutics aimed at reducing SOD1 aggregation. The identification of seeding-competent misfolded SOD1 in forms of ALS not associated with *SOD1* mutations may suggest a shared disease mechanism between familial and sporadic forms of ALS that could be targeted by SOD1-lowering therapies.

### Comparison of SOD1 seeding activity in cervical and thoracic spinal cords of sporadic ALS patients

A vast literature exists implicating SOD1 misfolding with *SOD1* fALS, and prion-like propagation of SOD1 misfolding in experimental models [4, 37]. SOD1 misfolding in sporadic ALS, as observed by immunohistochemistry and immunoprecipitation with SOD1 misfolding-selective antibodies, is more controversial [24]. Some studies show WT SOD1 can be induced to support prion-like propagation by transient expression of mutant SOD1 in cell culture [26] or mutant TDP-43 [45]. In these studies, WT SOD1 has been found to propagate from cell-to-cell, with blocking of transmission by the same misfolding-selective antibodies that detect misfolded SOD1 in sALS. However, in mouse models in vivo, and cell culture models in vitro, prion-like propagation of misfolded SOD1 seeds has not been detected to date from sALS tissue, despite the ready detection of SOD1 misfolding by *SOD1* fALS pathological material [5, 46]. Our current findings indicate SOD1 seeds are present in sALS cervical and thoracic spinal cords, and motor cortices, suggesting WT SOD1 can be misfolded to perform templating of WT rSOD1 substrate that is poorly structured (monomeric, one intramolecular disulfide bond, random coil, metal-free substrate). The different ThT fluorescence enhancements obtained by seeding fibrillization of this substrate with sALS cervical versus thoracic cord tissue suggests the seeds differ qualitatively in these distinct regions of sALS spinal cords. For example, these seeds may have different conformations that they imparted to some extent on the rSOD1 substrate molecules. ThT’s fluorescence yield is known to vary with the conformation of the amyloid fibrils to which it binds [52, 54]. However, at this point we cannot exclude the possibility that other factors besides seed conformation, such as seed concentration, average particle size, seed-associated ligands or cofactors, or tissue matrix components or contaminants (e.g., blood), might influence the relative RT-QuIC kinetics when seeded with sALS cervical cord, thoracic cord, and motor cortex tissue. The existence of different SOD1 seed conformations is supported by the observation of misfolded WT SOD1 being highly protease-sensitive [26] in contrast to fibrillized mutant SOD1 in *SOD1* fALS tissue [57].

### Comparison of SOD1 seeding activity in sALS, *SOD1* fALS, and *C9ORF72* fALS cord tissues

Several RT-QuIC assays, such as those for prion diseases and α-synucleinopathies, have shown a relationship between lag phase and protein seed concentration in neural tissues [28, 67], where lag phase extends as neural tissue is diluted. As expected, we see a similar relationship between ALS spinal neural tissue dilution and lag phase here, as well as a strong negative correlation between lag phase and 50% ThT fluorescence for *SOD1* fALS cervical cords (*R* = 0.88), *C9ORF72* fALS cervical cords (*R* = 0.85), and sporadic ALS thoracic cords (*R* = 0.84). In Fig. 6, the *SOD1* fALS cervical cords and sALS thoracic cords have higher 50% ThT fluorescence and stronger dependence on lag phase (i.e., more negative slope values) relative to cervical cords of sALS and *C9ORF72* patients, suggesting the former two ALS specimen types may have SOD1 seeds that better accommodate binding of ThT. As discussed in the previous section with respect to the ThT fluorescence differences seen between cervical and thoracic sALS cord tissue, such differences in seeding kinetics may reflect distinct seed conformations or influences of other potential components of the seeds or tissue specimens that vary between these ALS types. Further studies will be required to establish the basis and consistency of the differences we have observed in these initial analyses.

### SOD1 seeding activity between different anatomical regions within sporadic ALS patients

The observation of SOD1 seeding activity in primary medial motor cortex and thoracic spinal cord homogenates from five sporadic ALS patients suggests SOD1 seeding activity occurs in upper and lower motor neurons in sporadic ALS pathology (Fig. 5). Comparison of SOD1 seeding activity in each sporadic ALS patient’s motor cortex versus thoracic cord specimens indicates the motor cortices gave shortened lag phases relative to those given by thoracic spinal cord at equally matched dilutions (Fig. 7), suggesting potentially more SOD1 seeding activity in the primary medial motor cortex of these patients relative to their thoracic cord at the end stages of their disease. Further studies will be required to understand the prion-like spread of WT SOD1 in sporadic ALS pathology [59] and determining the extent of soluble versus insoluble misfolded SOD1 in patient neural tissues.

In conclusion, SAAs such as those on the RT-QuIC platform have provided ultrasensitive detection of specific pathological protein aggregates that cause disorders such as synucleinopathies and prion diseases [29, 43]. Our prototypic SOD1 RT-QuIC assay detects SOD1 seeds in ALS types that represent ≥ 95% of cases. On a fundamental level, our current findings provide new evidence for prion-like self-propagating assemblies of WT and mutant SOD1 in spinal cords and motor cortex of sporadic and familial ALS patients. Much additional investigation will be required to determine whether such seeding activity can be found in accessible biospecimens from living patients, and the extent to which abnormal forms of SOD1 with seeding activity are responsible for ALS pathogenesis. Analysis of SOD1 RT-QuIC kinetic parameters provides initial evidence that lag phase and 50% ThT correlate with SOD1 seed concentration and ALS specimen type. Thus, these RT-QuIC analyses may be useful in both basic research and potential biomarker applications.

### Supplementary Information

Below is the link to the electronic supplementary material.

Supplementary file1 (PDF 2511 KB)

## Data Availability

All data are included in supplementary information.
