# Supplementary material for: Seeding activity of human superoxide dismutase 1 aggregates in familial and sporadic amyotrophic lateral sclerosis postmortem neural tissues by real-time quaking-induced conversion
Source: Acta Neuropathol. 2024 Jun 17;147(1):100. doi: 10.1007/s00401-024-02752-8 (PMC11182821; doi:10.1007/s00401-024-02752-8)
Supplement: Supplementary file 1 — Supplementary file1 (PDF 2511 KB) [file 401_2024_2752_MOESM1_ESM.pdf]

Supporting Information

Seeding Activity of Human Superoxide Dismutase 1 Aggregates in Familial and Sporadic Amyotrophic Lateral Sclerosis Postmortem Neural Tissues By Real-Time Quaking-Induced Conversion

Justin K. Mielke[1], Mikael Klingeborn[1], Eric P. Schultz[2], Erin L. Markham[1], Emily D. Reese[1*], Parvez Alam[3*], Ian R. Mckenzie[4], Cindy V. Ly[5], Byron Caughey[3], Neil R. Cashman[4], Moses J. Leavens[1]

[1]McLaughlin Research Institute for Biomedical Sciences, Great Falls, Montana, USA

[2]Center for Biomolecular Structure and Dynamics, University of Montana, Missoula, Montana, USA

[3]Laboratory of Neurological Infections and Immunity, Rocky Mountain Laboratories, National Institute of Allergy and Infectious Diseases, National Institutes of Health, Hamilton, Montana, USA

[4]Departments of Pathology and Medicine, University of British Columbia, Vancouver, BC, Canada

[5]Department of Neurology, Washington University, Saint Louis, Missouri, USA

*authors contributed equally

To whom correspondence should be addressed: email: mleavens@mclaughlinresearch.org

Table S1. Human non-mutant SOD1 cDNA and amino acid sequence information.

5’-
tggcgaatgggacgcgccctgtagcggcgcattaagcgcggcgggtgtggtggttacgcgcagcgtgaccgctacacttgccagcgccctagcgcccgctcct
ttcgctttcttcccttcctttctcgccacgttcgccggctttccccgtcaagctctaaatcgggggctccctttagggttccgatttagtgctttacggcacctcgacccca
aaaaacttgattagggtgatggttcacgtagtgggccatcgccctgatagacggtttttcgccctttgacgttggagtccacgttctttaatagtggactcttgttccaaa
ctggaacaacactcaaccctatctcggtctattcttttgatttataagggattttgccgatttcggcctattggttaaaaaatgagctgatttaacaaaaatttaacgcgaat
tttaacaaaatattaacgtttacaatttcaggtggcacttttcggggaaatgtgcgcggaacccctatttgtttatttttctaaatacattcaaatatgtatccgctcatgaatt
aattcttagaaaaactcatcgagcatcaaatgaaactgcaatttattcatatcaggattatcaataccatatttttgaaaaagccgtttctgtaatgaaggagaaaactca
ccgaggcagttccataggatggcaagatcctggtatcggtctgcgattccgactcgtccaacatcaatacaacctattaatttcccctcgtcaaaaataaggttatcaa
gtgagaaatcaccatgagtgacgactgaatccggtgagaatggcaaaagtttatgcatttctttccagacttgttcaacaggccagccattacgctcgtcatcaaaat
cactcgcatcaaccaaaccgttattcattcgtgattgcgcctgagcgagacgaaatacgcgatcgctgttaaaaggacaattacaaacaggaatcgaatgcaaccg
gcgcaggaacactgccagcgcatcaacaatattttcacctgaatcaggatattcttctaatacctggaatgctgttttcccggggatcgcagtggtgagtaaccatgc
atcatcaggagtacggataaaatgcttgatggtcggaagaggcataaattccgtcagccagtttagtctgaccatctcatctgtaacatcattggcaacgctacctttg
ccatgtttcagaaacaactctggcgcatcgggcttcccatacaatcgatagattgtcgcacctgattgcccgacattatcgcgagcccatttatacccatataaatcag
catccatgttggaatttaatcgcggcctagagcaagacgtttcccgttgaatatggctcataacaccccttgtattactgtttatgtaagcagacagttttattgttcatga
ccaaaatcccttaacgtgagttttcgttccactgagcgtcagaccccgtagaaaagatcaaaggatcttcttgagatcctttttttctgcgcgtaatctgctgcttgcaaa
caaaaaaaccaccgctaccagcggtggtttgtttgccggatcaagagctaccaactctttttccgaaggtaactggcttcagcagagcgcagataccaaatactgtc
cttctagtgtagccgtagttaggccaccacttcaagaactctgtagcaccgcctacatacctcgctctgctaatcctgttaccagtggctgctgccagtggcgataagt
cgtgtcttaccgggttggactcaagacgatagttaccggataaggcgcagcggtcgggctgaacggggggttcgtgcacacagcccagcttggagcgaacgac
ctacaccgaactgagatacctacagcgtgagctatgagaaagcgccacgcttcccgaagggagaaaggcggacaggtatccggtaagcggcagggtcggaac
aggagagcgcacgagggagcttccagggggaaacgcctggtatctttatagtcctgtcgggtttcgccacctctgacttgagcgtcgatttttgtgatgctcgtcag
gggggcggagcctatggaaaaacgccagcaacgcggcctttttacggttcctggccttttgctggccttttgctcacatgttctttcctgcgttatcccctgattctgtg
gataaccgtattaccgcctttgagtgagctgataccgctcgccgcagccgaacgaccgagcgcagcgagtcagtgagcgaggaagcggaagagcgcctgatg
cggtattttctccttacgcatctgtgcggtatttcacaccgcatatatggtgcactctcagtacaatctgctctgatgccgcatagttaagccagtatacactccgctatc
gctacgtgactgggtcatggctgcgccccgacacccgccaacacccgctgacgcgccctgacgggcttgtctgctcccggcatccgcttacagacaagctgtga
ccgtctccgggagctgcatgtgtcagaggttttcaccgtcatcaccgaaacgcgcgaggcagctgcggtaaagctcatcagcgtggtcgtgaagcgattcacaga
tgtctgcctgttcatccgcgtccagctcgttgagtttctccagaagcgttaatgtctggcttctgataaagcgggccatgttaagggcggtttttcctgtttggtcactga
tgcctccgtgtaagggggatttctgttcatgggggtaatgataccgatgaaacgagagaggatgctcacgatacgggttactgatgatgaacatgcccggttactgg
aacgttgtgagggtaaacaactggcggtatggatgcggcgggaccagagaaaaatcactcagggtcaatgccagcgcttcgttaatacagatgtaggtgttccac
agggtagccagcagcatcctgcgatgcagatccggaacataatggtgcagggcgctgacttccgcgtttccagactttacgaaacacggaaaccgaagaccatt
catgttgttgctcaggtcgcagacgttttgcagcagcagtcgcttcacgttcgctcgcgtatcggtgattcattctgctaaccagtaaggcaaccccgccagcctagc
cgggtcctcaacgacaggagcacgatcatgcgcacccgtggggccgccatgccggcgataatggcctgcttctcgccgaaacgtttggtggcgggaccagtga
cgaaggcttgagcgagggcgtgcaagattccgaataccgcaagcgacaggccgatcatcgtcgcgctccagcgaaagcggtcctcgccgaaaatgacccaga
gcgctgccggcacctgtcctacgagttgcatgataaagaagacagtcataagtgcggcgacgatagtcatgccccgcgcccaccggaaggagctgactgggtt
gaaggctctcaagggcatcggtcgagatcccggtgcctaatgagtgagctaacttacattaattgcgttgcgctcactgcccgctttccagtcgggaaacctgtcgt
gccagctgcattaatgaatcggccaacgcgcggggagaggcggtttgcgtattgggcgccagggtggtttttcttttcaccagtgagacgggcaacagctgattgc
ccttcaccgcctggccctgagagagttgcagcaagcggtccacgctggtttgccccagcaggcgaaaatcctgtttgatggtggttaacggcgggatataacatg
agctgtcttcggtatcgtcgtatcccactaccgagatatccgcaccaacgcgcagcccggactcggtaatggcgcgcattgcgcccagcgccatctgatcgttggc
aaccagcatcgcagtgggaacgatgccctcattcagcatttgcatggtttgttgaaaaccggacatggcactccagtcgccttcccgttccgctatcggctgaatttg
attgcgagtgagatatttatgccagccagccagacgcagacgcgccgagacagaacttaatgggcccgctaacagcgcgatttgctggtgacccaatgcgacca
gatgctccacgcccagtcgcgtaccgtcttcatgggagaaaataatactgttgatgggtgtctggtcagagacatcaagaaataacgccggaacattagtgcaggc
agcttccacagcaatggcatcctggtcatccagcggatagttaatgatcagcccactgacgcgttgcgcgagaagattgtgcaccgccgctttacaggcttcgacg
ccgcttcgttctaccatcgacaccaccacgctggcacccagttgatcggcgcgagatttaatcgccgcgacaatttgcgacggcgcgtgcagggccagactgga
ggtggcaacgccaatcagcaacgactgtttgcccgccagttgttgtgccacgcggttgggaatgtaattcagctccgccatcgccgcttccactttttcccgcgttttc
gcagaaacgtggctggcctggttcaccacgcgggaaacggtctgataagagacaccggcatactctgcgacatcgtataacgttactggtttcacattcaccaccc
tgaattgactctcttccgggcgctatcatgccataccgcgaaaggttttgcgccattcgatggtgtccgggatctcgacgctctcccttatgcgactcctgcattagga
agcagcccagtagtaggttgaggccgttgagcaccgccgccgcaaggaatggtgcatgcaaggagatggcgcccaacagtcccccggccacggggcctgcc
accatacccacgccgaaacaagcgctcatgagcccgaagtggcgagcccgatcttccccatcggtgatgtcggcgatataggcgccagcaaccgcacctgtgg

cgccggtgatgccggccacgatgcgtccggcgtagaggatcgagatctcgatcccgcgaaattaatacgactcactataggggaattgtgagcggataacaattc
ccctctagaaataattttgtttaactttaagaaggagatataccatgcatcatcatcatcatcacagcagcggcctggtgccgcgcggcagccatatggcaacaaaa
gctgtatgtgttctaaagggcgatggtccggttcaaggtatcatcaacttcgagcagaaagaatctaatggcccggtcaaggtgtggggttcgatcaaaggtttgac
cgaaggtttacatggttttcatgttcacgagttcggcgacaacaccgcgggctgcacgtctgcaggcccacactttaacccgctgtcccgcaagcacggcggtcc
gaaagatgaagaacgtcatgtgggtgacctgggcaatgttactgcggacaaggacggcgtggccgacgttagcattgaagatagcgttattagcctgagcggtg
accactgcattattggtcgtaccttggtcgtgcacgagaaggcggatgatctgggtaaaggcggaaacgaggagtccaccaaaaccggtaatgctggcagccgt
ctggcatgtggtgtgatcggcatcgcgcagtaactcgagcaccaccaccaccaccactgagatccggctgctaacaaagcccgaaaggaagctgagttggctgc
tgccaccgctgagcaataactagcataaccccttggggcctctaaacgggtcttgaggggttttttgctgaaaggaggaactatatccggat-3’

<u>Amino acid sequence (underline is tag)</u>
N-terminus-
<u>MHHHHHHSSGLVPRGSH</u>MATKAVCVLKGDGPVQGIINFEQKESNGPVKVWGSIKGLTEGLHGFHVHE
FGDNTAGCTSAGPHFNPLSRKHGGPKDEERHVGDLGNVTADKDGVADVSIEDSVISLSGDHCIIGRTLV
VHEKADDLGKGGNEESTKTGNAGSRLACGVIGIAQ**-C terminus**

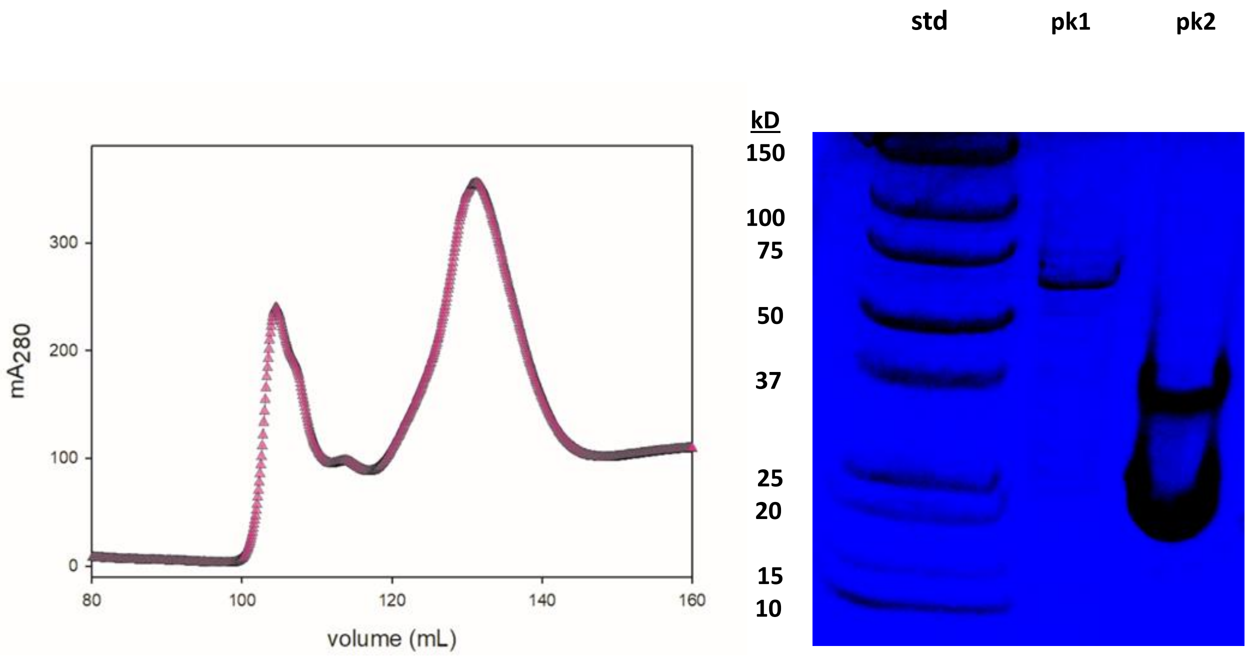

Figure S1. Fast protein liquid chromatogram of BL21 *E. coli* expressed human SOD1 WT eluted via an imidazole gradient. The later eluting peak, peak 2 (pk2, 120-140 mL), is human SOD1 WT (Left), determined via SDS-PAGE (no heat, no reducing agent) stained with Coomassie (Right), showing pk2 is in dimer and monomer equilibrium. Peak 2 was further characterized to confirm mass and identity.

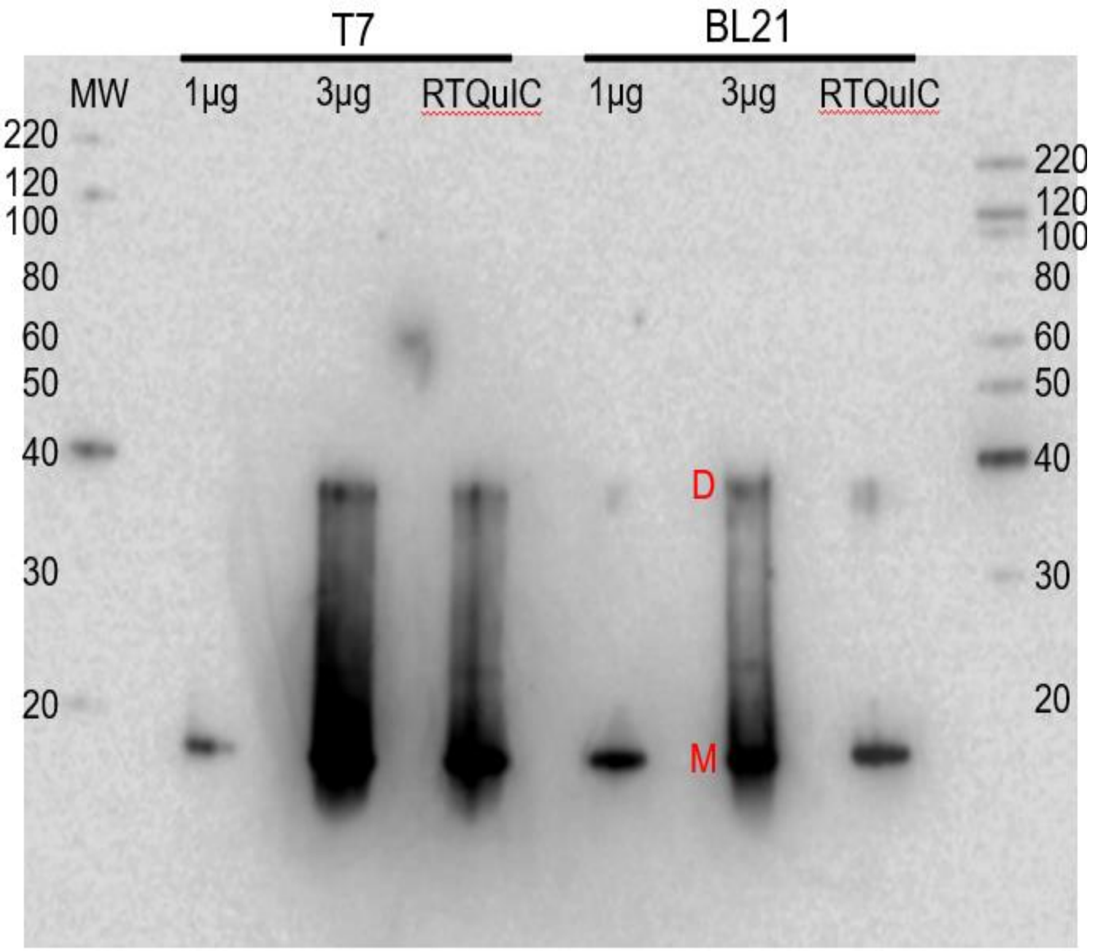

Figure S2. Native PAGE immunoblot of T7 or BL21 *E. coli* expressed human SOD1 WT purified with β-ME that is subsequently exchanged into 25 mM Tris pH 8.0 (1 or 3 µg lanes). For human SOD1 WT expressed from BL21 *E. coli*, exchange of 3 µg from 25 mM Tris pH 8.0 into RT-QuIC buffer shifts equilibrium from dimer/monomer population to primarily monomer population. Dimer is represented as 'D' and monomer is represented as 'M'. Human SOD1 WT in our RT-QuIC buffer was used as a substrate in the RT-QuIC assay. Apparent molecular weights in kDa are indicated on the left- and right-hand sides alongside a molecular weight standard (MW).

A

B

C

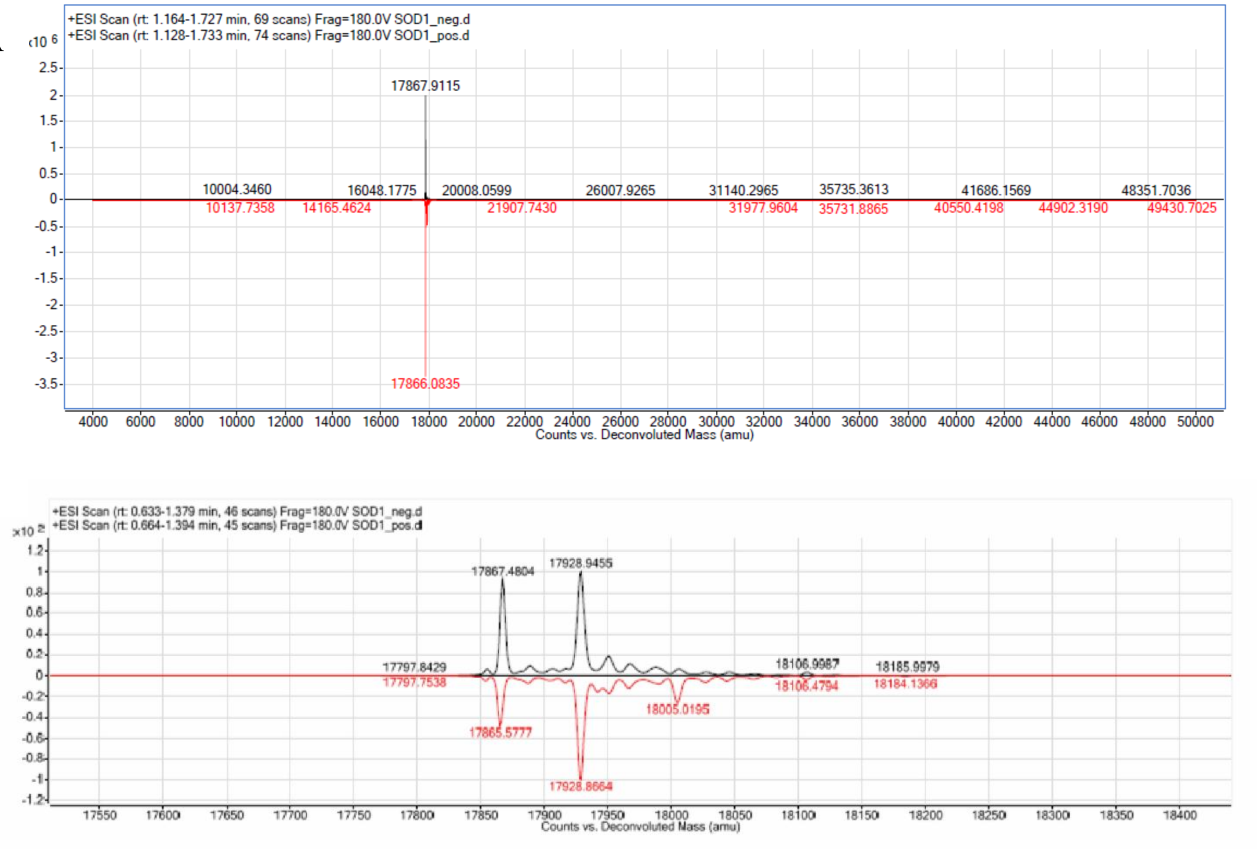

Figure S3. Electrospray ionization LC-MS mass spectrum mirror plot of purified SOD1 WT in 25 mM Tris pH 8.0. (**A**) SOD1 WT not treated with β-ME (**black**). (B) SOD1 WT treated with β-ME (red). Expected theoretical mass is 17,867.86 atomic mass unit (from ExPASy Peptide Mass tool (http://web.expasy.org/peptide_mass/) using average mass, with no cutting and $[M+H]^+$ options). The 2 Da mass shift in β-ME-treated SOD1 WT (red) indicates the presence of an intramolecular disulfide bond [45]. Experiments were run at ~pH 2.8. (**C**) Electrospray ionization LC-MS mass spectrum of SOD1 WT at pH ~5.5 indicating copper bound to both SOD1 WT preparations (treated or not treated with β-ME).

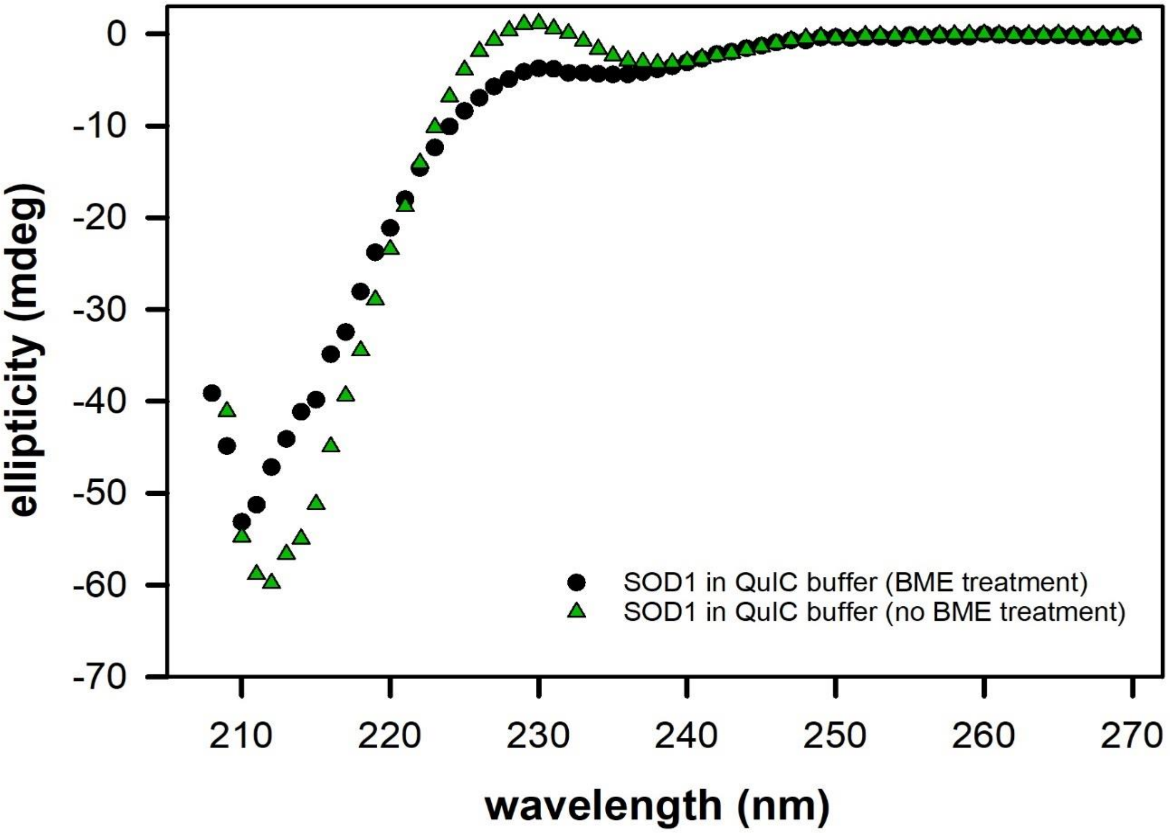

Figure S4. CD spectroscopy of mean wavelength scans in the far UV region with 50 µM SOD1 WT purified in the presence or absence of β-ME. Both β-ME-treated SOD1 WT or non-treated SOD1 WT were exchanged into a Tris buffer pH 8.0 (see **Fig. S2**) then put in RT-QuIC buffer. Only recombinant SOD1 WT treated with β-ME during purification was an efficient SOD1 substrate for SOD1 RT-QuIC. The decrease at 225-230 nm is attributable to a loss of polyproline II helix secondary structure [46–48]. The loss of polyproline II helix structure indicates a monomeric random coil for β-ME treated human SOD1 WT. These conditions likely are a thermodynamic requirement for SOD1 WT to be an effective substrate for SOD1 RT-QuIC, as non-reduced SOD1 WT substrate does not convert.

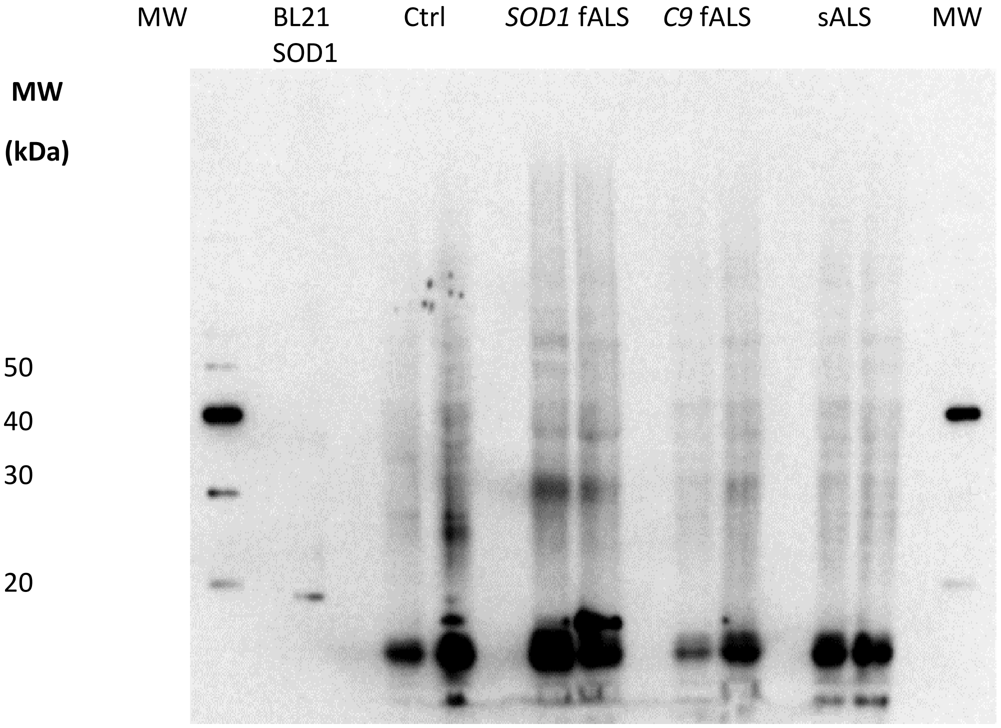

Figure S5. Native PAGE immunoblot for SOD1 of postmortem human ALS patient spinal cord homogenates and human negative control spinal cord homogenates. 0.05 µg of purified BL21 SOD1 WT (BL21 SOD1). 1.93 µg of total protein from spinal cord homogenates of two donors each of negative controls (Ctrl), *SOD1* fALS, *C9ORF72* fALS, and sALS, are indicated above the blot. Apparent molecular weights in kDa are indicated on the left- and right-hand sides alongside a molecular weight standard (MW).

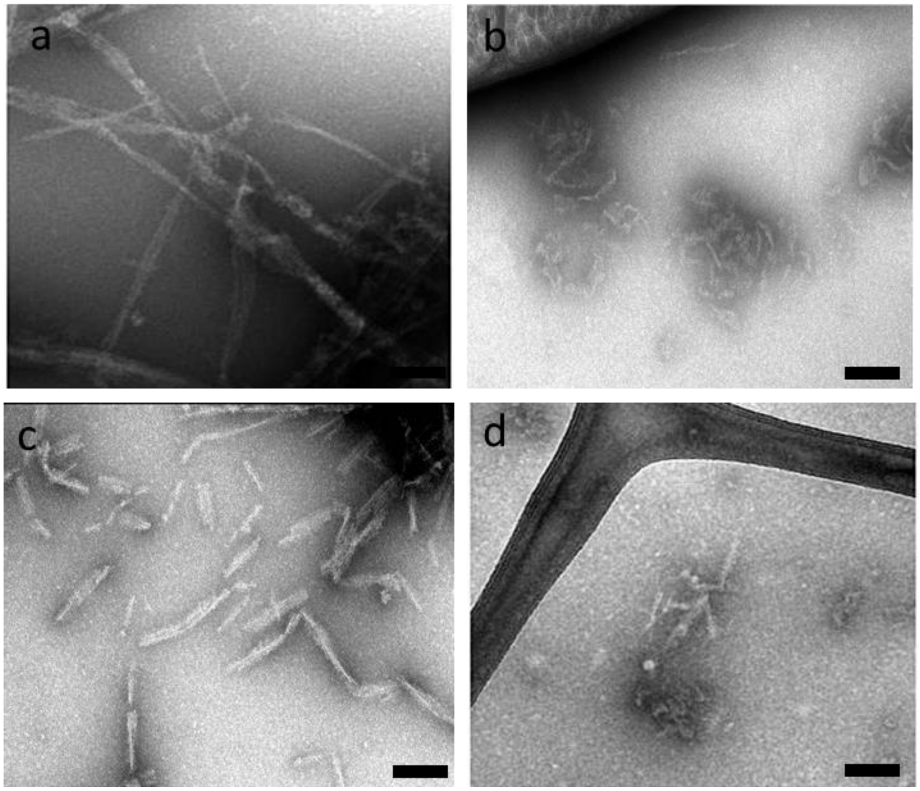

Figure S6. Representative electron micrograph of SOD1 RT- QuIC end products. (**a**) Familial ALS patient with abnormal *C9ORF72* expansion, (**b**) *SOD1* fALS patient 1, (**c**) Sporadic ALS patient 5, and (**d**) negative control patient 1. All reactions were seeded with 10^{-4} dilution of spinal cord. Scale bar = 200 nm.

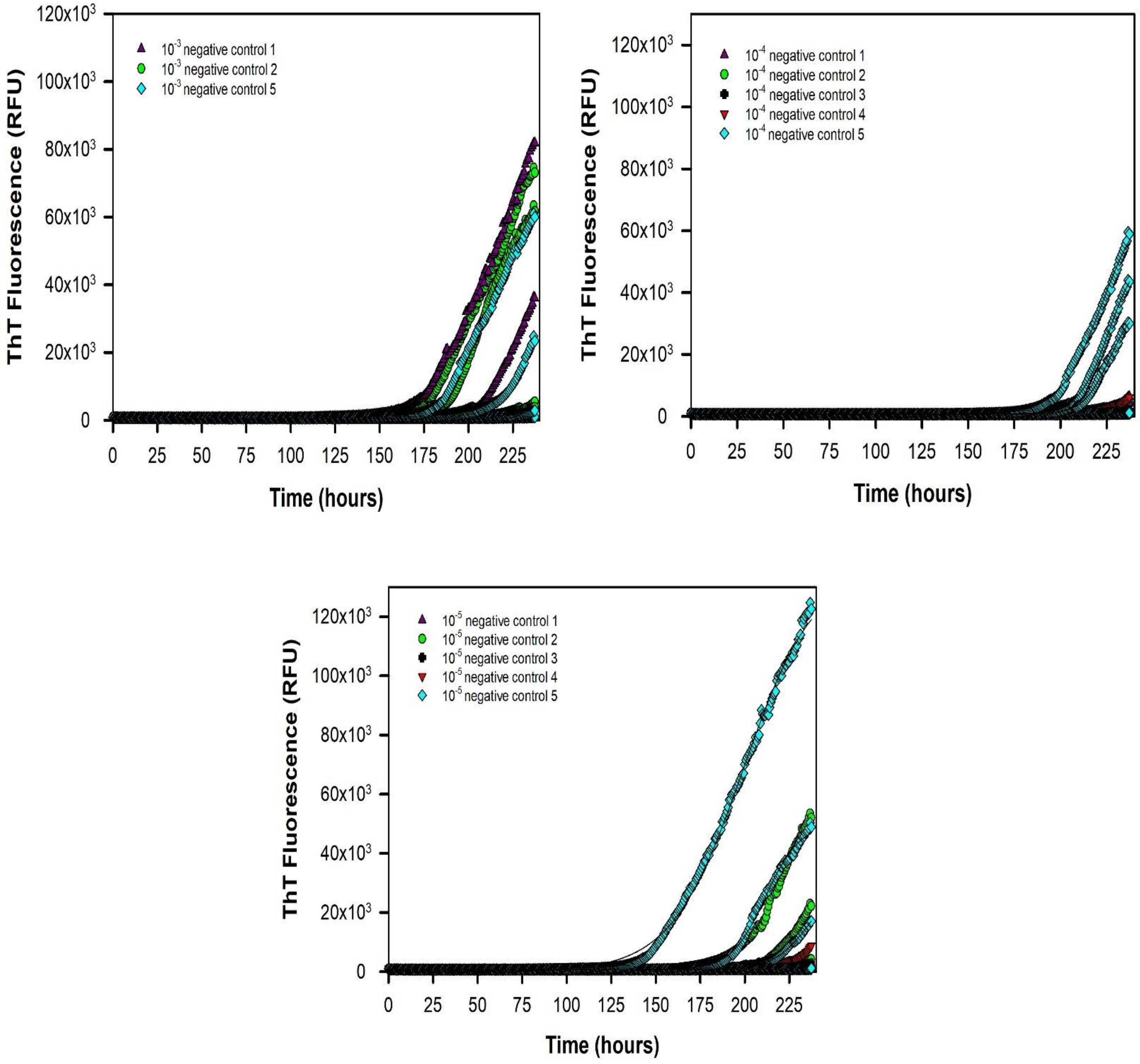

Figure S7. ThT fluorescence (RFU) versus time (hours) for negative control thoracic spinal cord homogenates run in SOD1 RT-QuIC assay from 10^{-3} to 10^{-5} dilution.

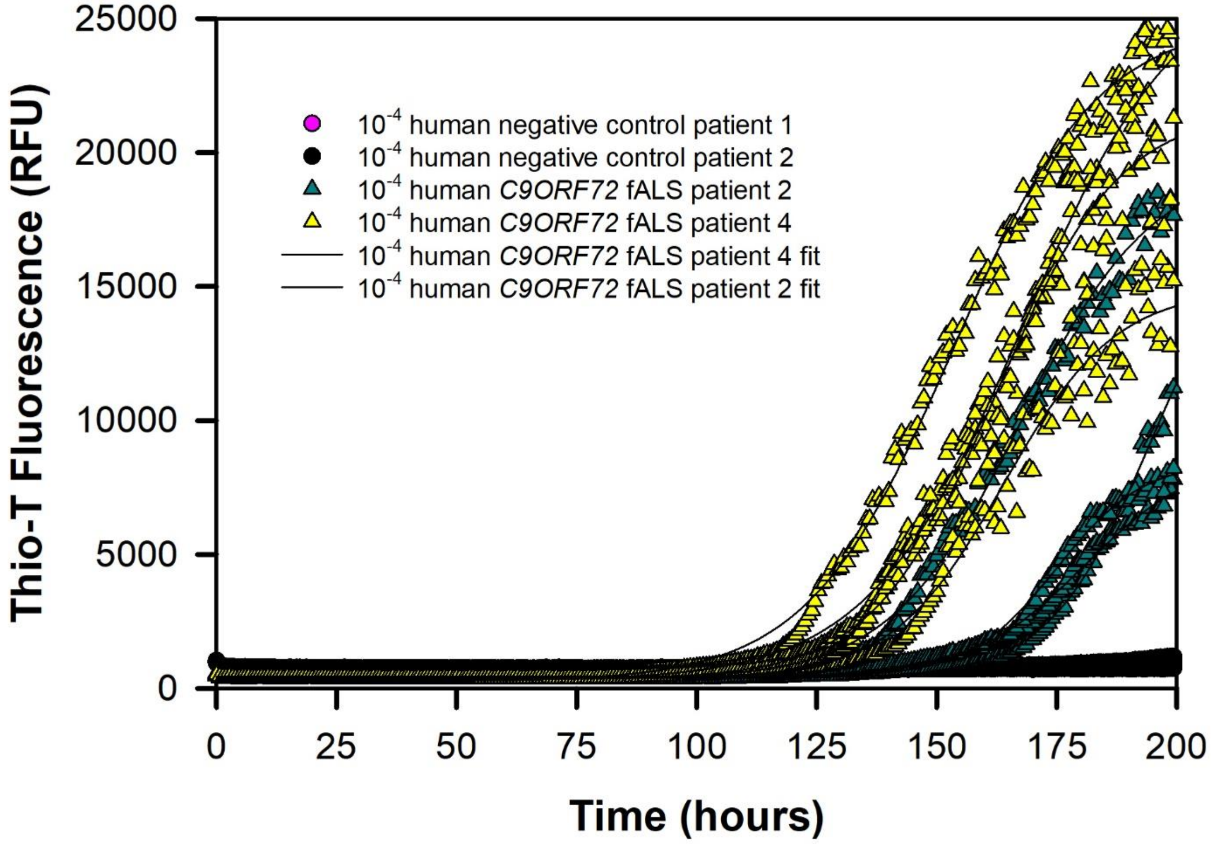

Figure S8. Kinetics of SOD1 RT-QuIC data from ALS spinal cord homogenates at 10^{-4} dilution. Solid curves are fits of the data to a sigmoidal equation.

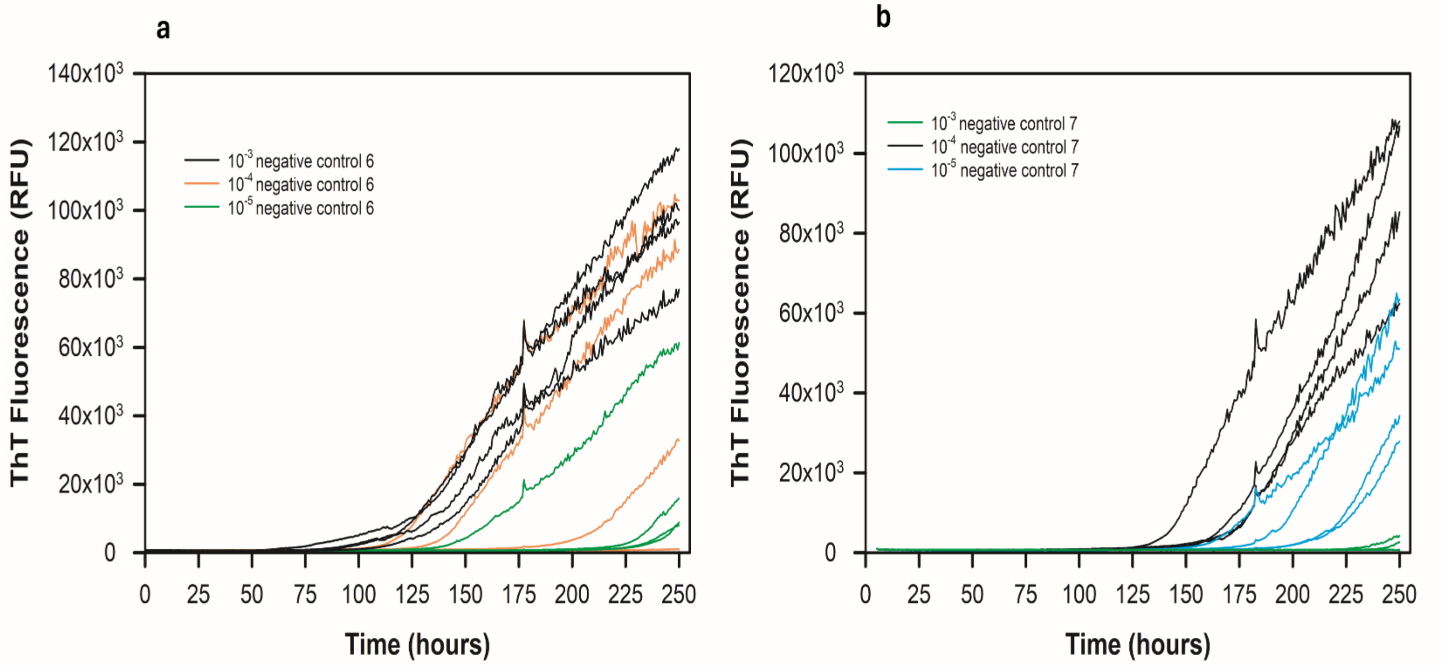

Figure S9. ThT fluorescence (RFU) versus time (hours) for negative control 6 (a) and 7 (b) cervical spinal cord homogenates run in SOD1 RT-QuIC assay from 10^{-3} to 10^{-5} dilution.

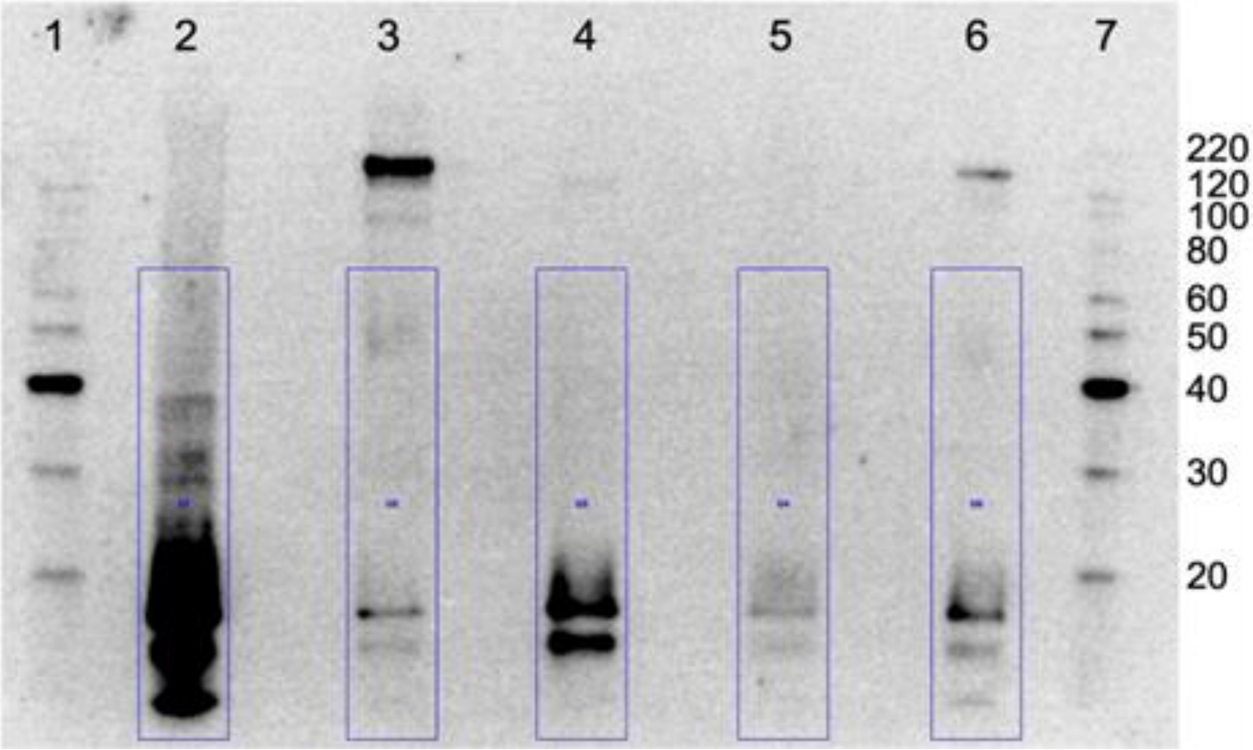

Figure S10. Immunoprecipitation of sALS patient 4 spinal cord homogenate flow-through after antibody (Ab) capture. Lanes 1 and 7 are molecular weight standards, lane 2 is 5 µL of 10% w/v sALS cord input (100%), sALS flow through after Ab capture is shown in lanes 3 (pan-SOD1 Ab, 23%), 4 (pan-SOD1 isotype control, 75%), 5 (C4F6, 21%), and 6 (C4F6 isotype control, 28%). Blue boxes are quantification areas to assess immunodepletion. Percent number is amount remaining SOD1 compared to input. Apparent molecular weights in kDa are indicated on the right-hand side.

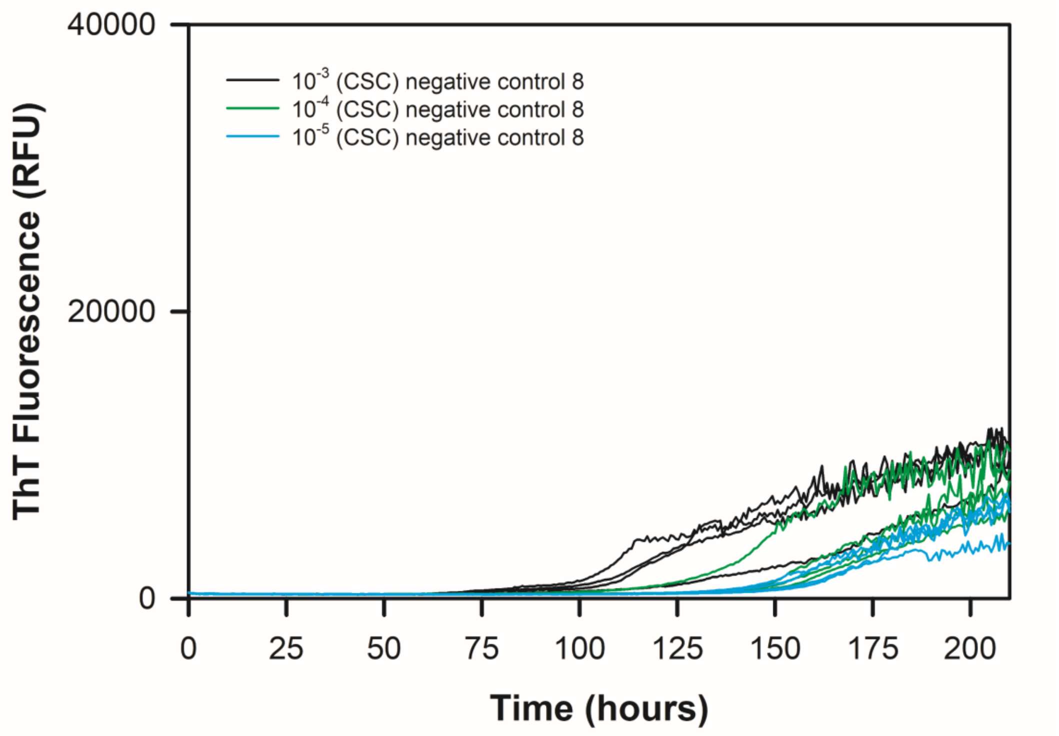

Figure S11. ThT fluorescence (RFU) versus time for cervical spinal cord (CSC) negative control 8, run in the SOD1 RT-QuIC assay from 10^{-3} to 10^{-5} dilution.

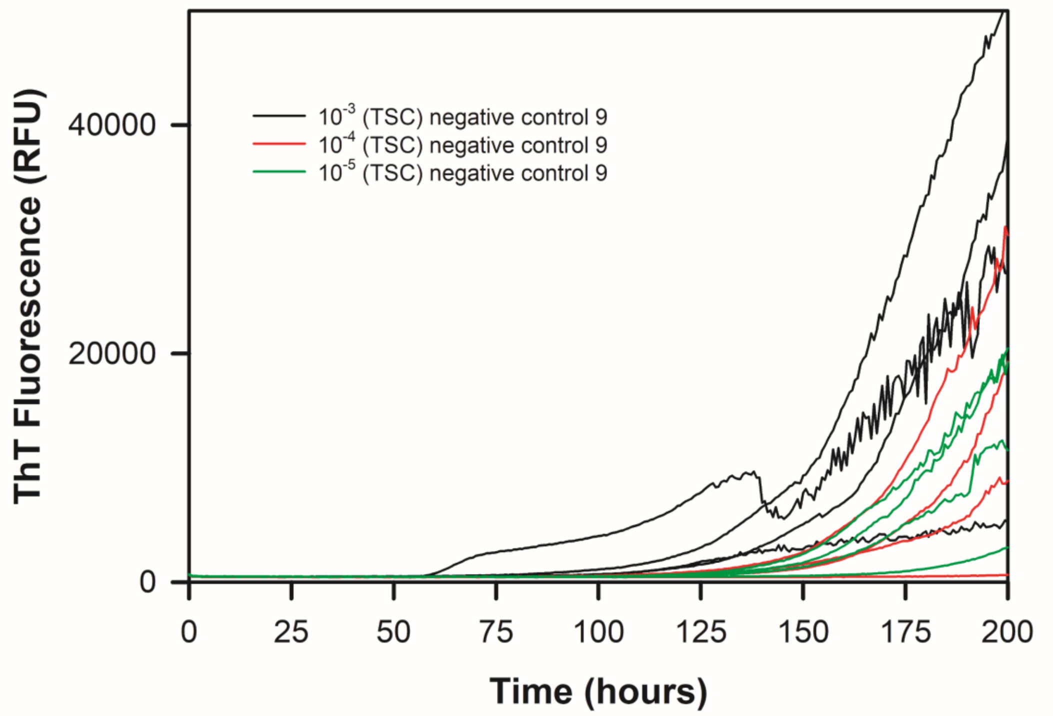

Figure S12. ThT fluorescence (RFU) versus time for thoracic spinal cord (TSC) negative control 9 run in SOD1 RT-QuIC assay from 10^{-3} to 10^{-5} dilution.

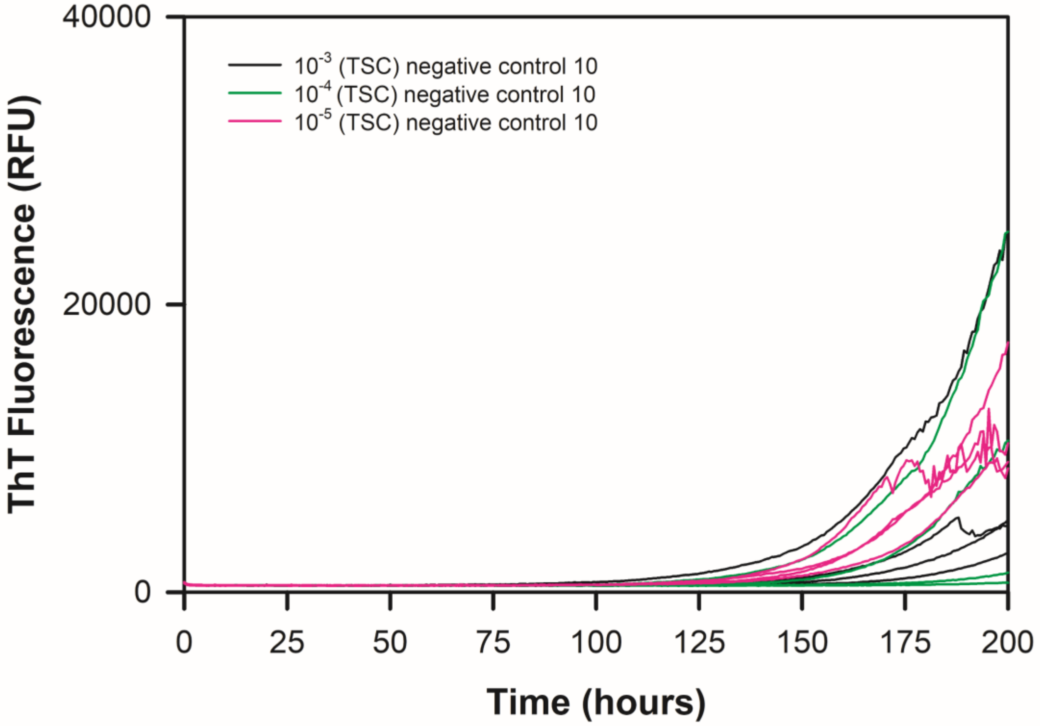

Figure S13. ThT fluorescence (RFU) versus time for thoracic spinal cord (TSC) negative control 10 run in SOD1 RT-QuIC assay from 10^{-3} to 10^{-5} dilution.

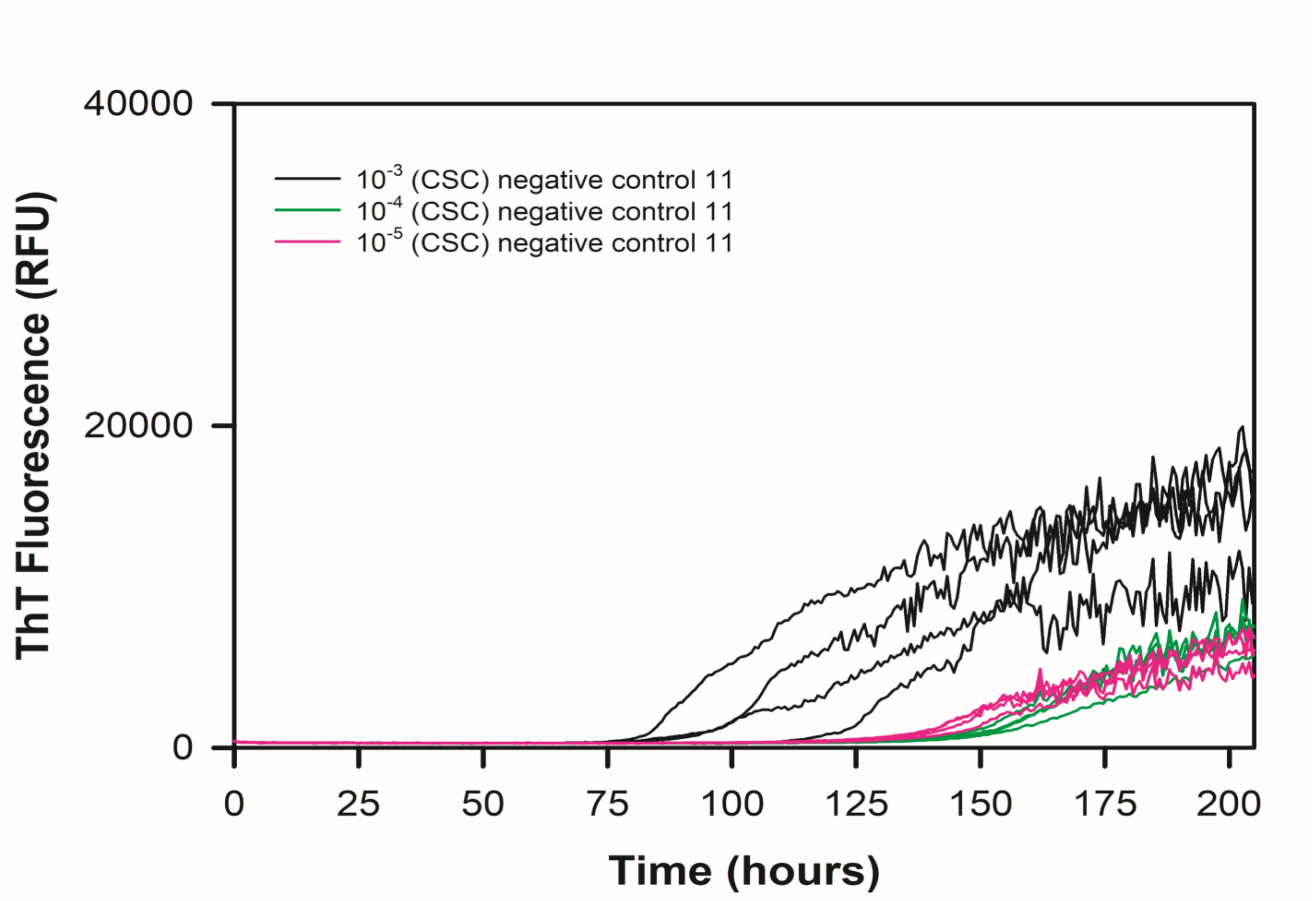

Figure S14. ThT fluorescence (RFU) versus time for cervical spinal cord (CSC) negative control 11 run in SOD1 RT-QuIC assay from 10^{-3} to 10^{-5} dilution.

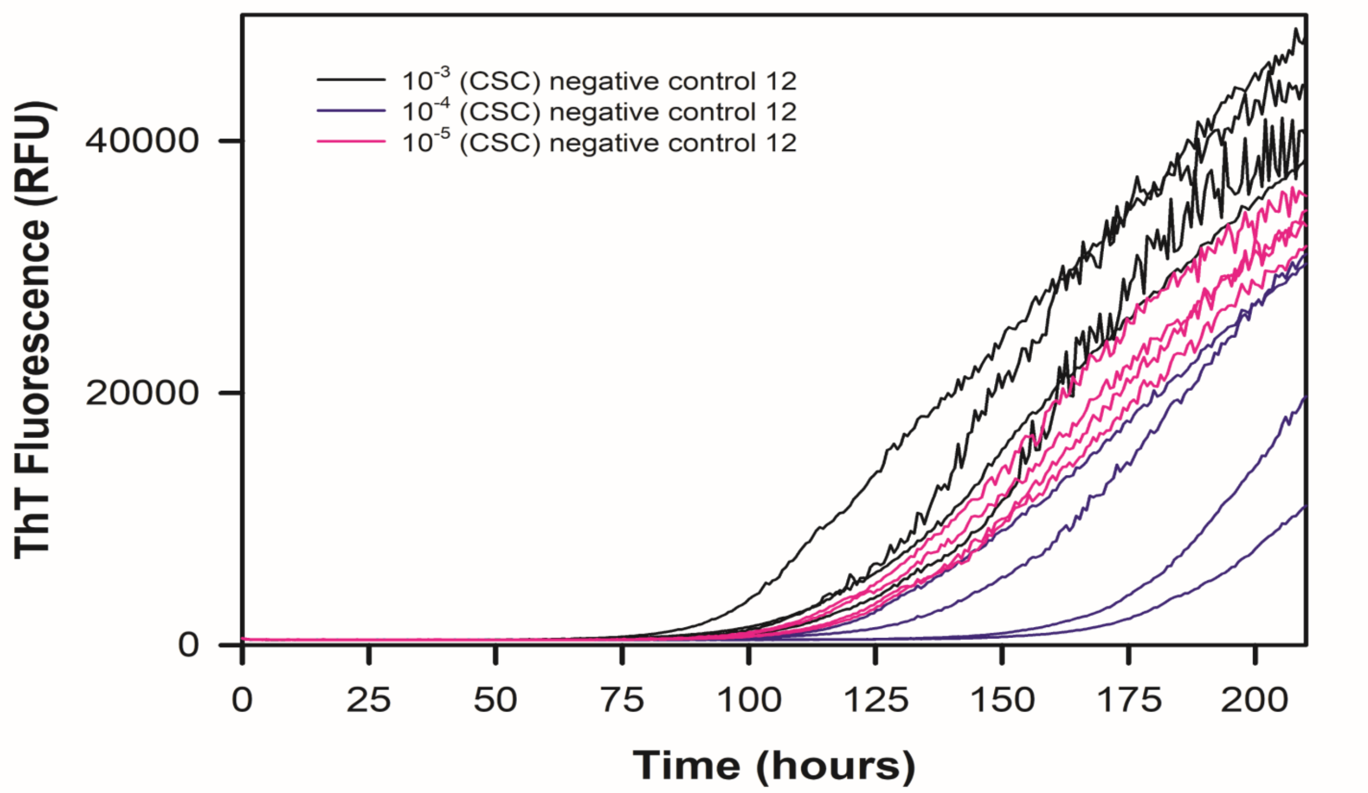

Figure S15. ThT fluorescence (RFU) versus time for cervical spinal cord (CSC) negative control 12 run in SOD1 RT-QuIC assay from 10^{-3} to 10^{-5} dilution.

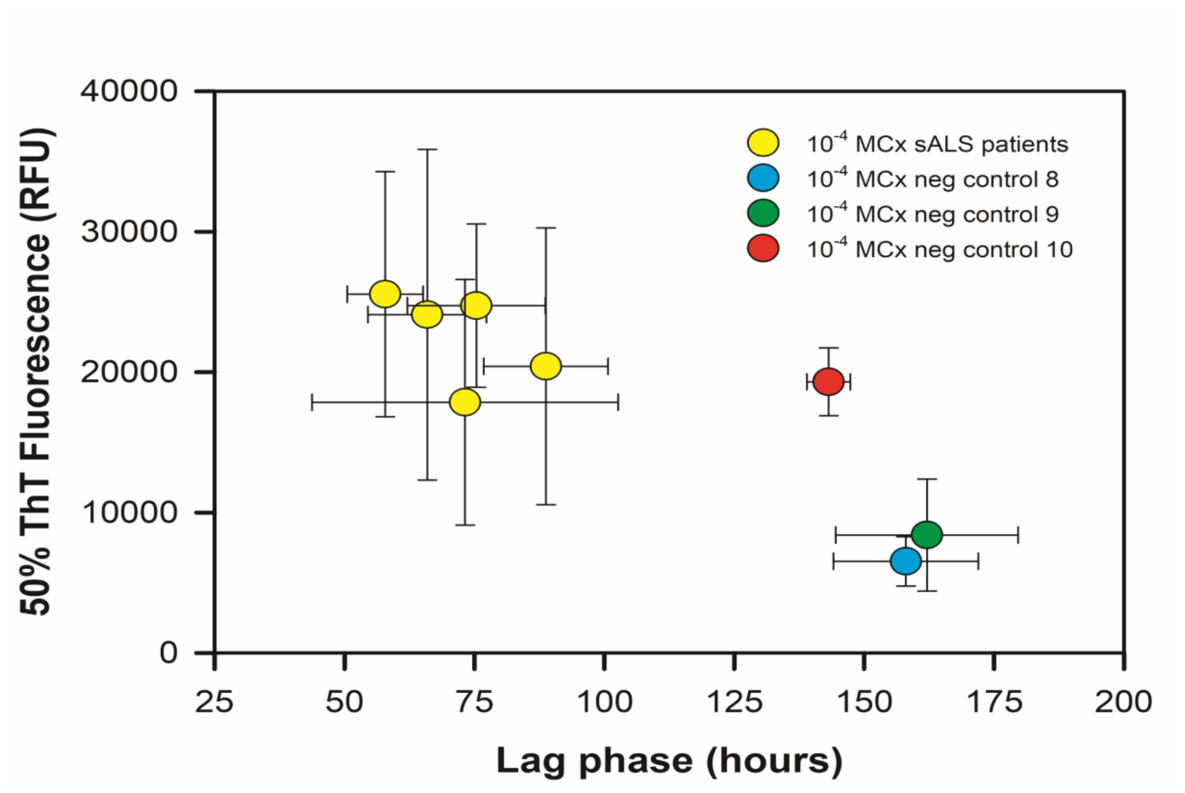

Figure S16. 50% ThT fluorescence (RFU) versus lag phase (hours) of SOD1 seeding activity in motor cortex from sporadic ALS patients and other non-ALS neurological disease controls.

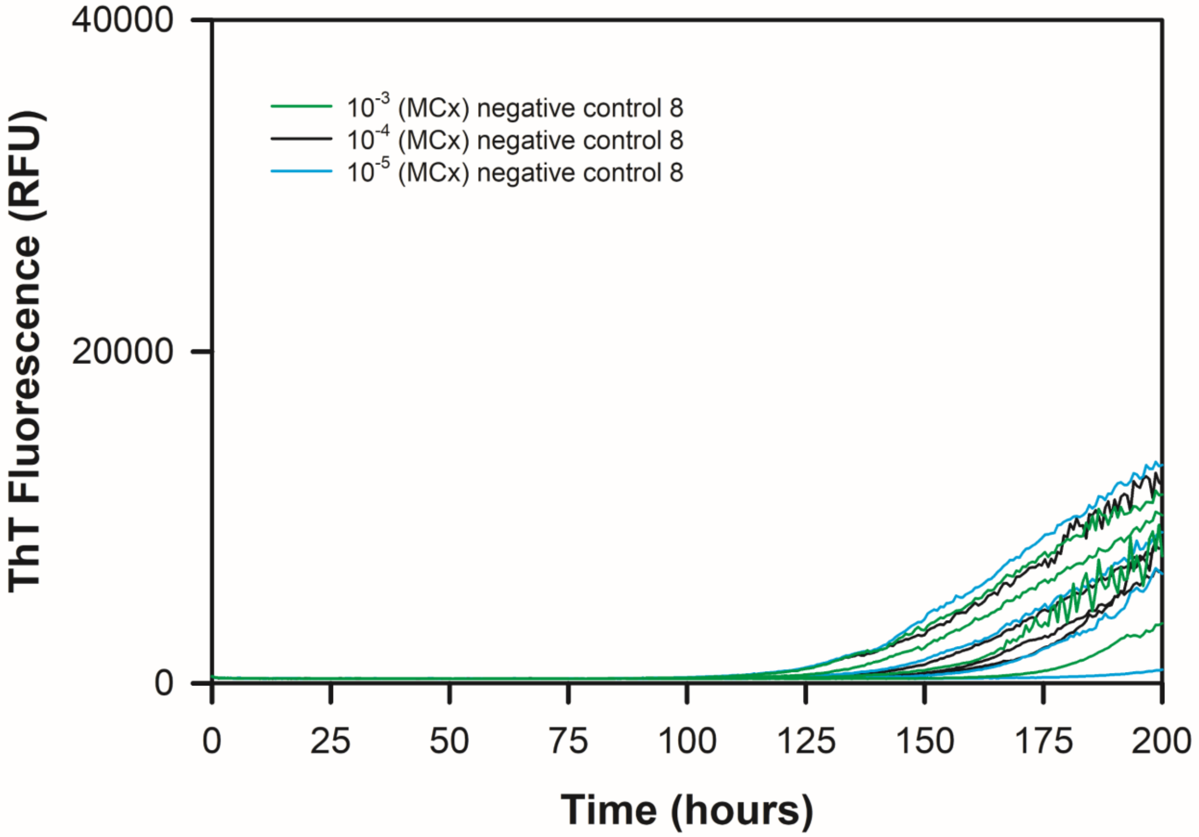

Figure S17. ThT fluorescence (RFU) versus time for motor cortex (MCx) negative control 8 run in SOD1 RT-QuIC assay from 10^{-3} to 10^{-5} dilution.

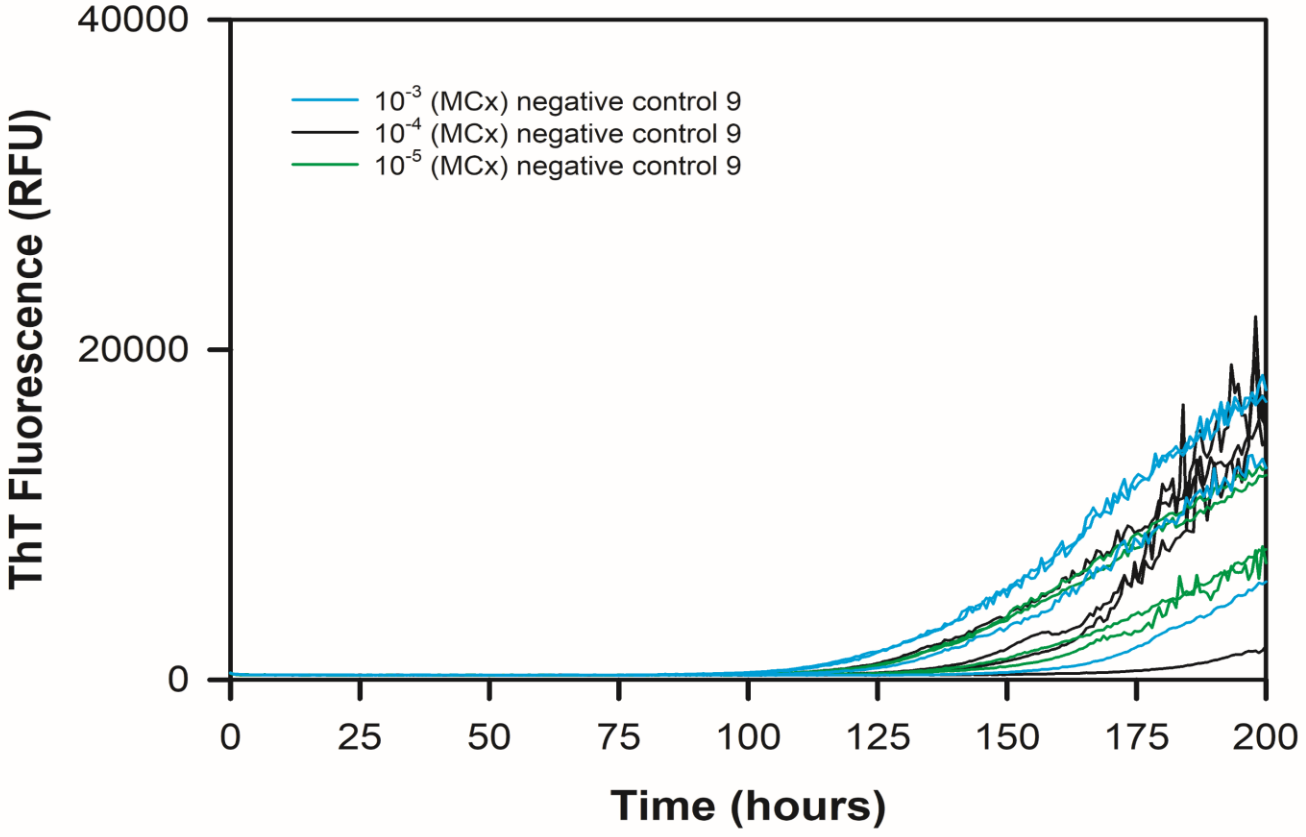

Figure S18. ThT fluorescence (RFU) versus time for motor cortex (MCx) negative control 9 run in SOD1 RT-QuIC assay from 10^{-3} to 10^{-5} dilution.

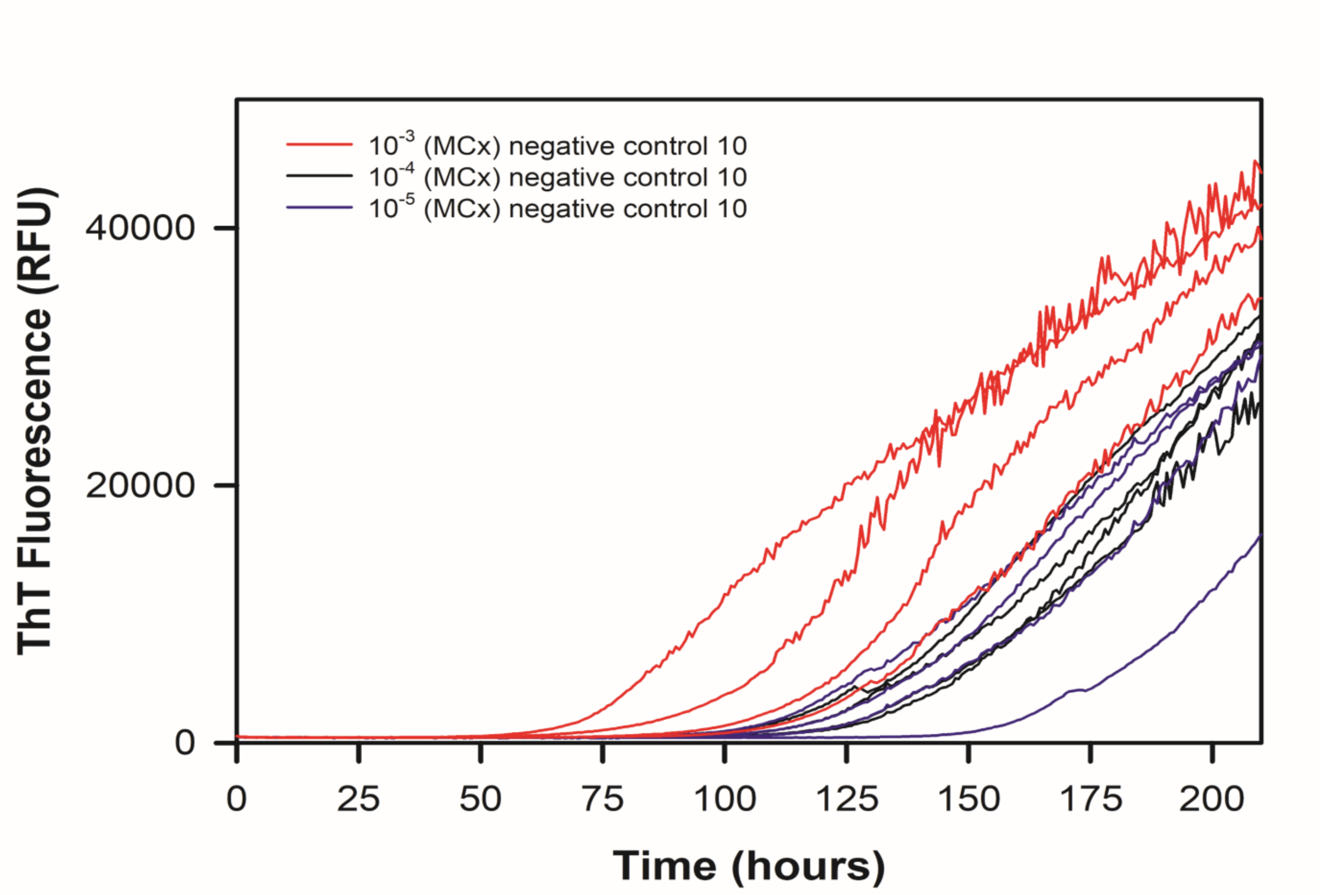

Figure S19. ThT fluorescence (RFU) versus time for motor cortex (MCx) negative control 10 run in SOD1 RT-QuIC assay from 10^{-3} to 10^{-5} dilution.
